# Zika virus exacerbates encephalomyelitis by inducing the production of T cell-attracting chemokines in astrocytes

**DOI:** 10.1093/intimm/dxaf075

**Published:** 2025-12-17

**Authors:** Naganori Kamiyama, Benjawan Saechue, Nozomi Sachi, Thanyakorn Chalalai, Astri Dewayani, Masaaki Okamoto, Sotaro Ozaka, Yasuhiro Soga, Yomei Kagoshima, Supanuch Ekronarongchai, Shinya Hidano, Makoto Tsuda, Takashi Kobayashi

**Affiliations:** Department of Infectious Disease Control, Faculty of Medicine, Oita University, Oita 879-5593, Japan; Research Center for GLOBAL and LOCAL Infectious Diseases, Oita University, Oita 879-5593, Japan; Department of Infectious Disease Control, Faculty of Medicine, Oita University, Oita 879-5593, Japan; Department of Infectious Disease Control, Faculty of Medicine, Oita University, Oita 879-5593, Japan; Department of Infectious Disease Control, Faculty of Medicine, Oita University, Oita 879-5593, Japan; Department of Infectious Disease Control, Faculty of Medicine, Oita University, Oita 879-5593, Japan; Department of Infectious Disease Control, Faculty of Medicine, Oita University, Oita 879-5593, Japan; Department of Infectious Disease Control, Faculty of Medicine, Oita University, Oita 879-5593, Japan; Department of Infectious Disease Control, Faculty of Medicine, Oita University, Oita 879-5593, Japan; Department of Infectious Disease Control, Faculty of Medicine, Oita University, Oita 879-5593, Japan; Department of Infectious Disease Control, Faculty of Medicine, Oita University, Oita 879-5593, Japan; Department of Immune Regulation, The Research Center for Hepatitis and Immunology, National Center for Global Health and Medicine, Chiba 272-8516, Japan; Department of Molecular and System Pharmacology, Graduate School of Pharmaceutical Sciences, Kyushu University, Fukuoka 812-8582, Japan; Department of Infectious Disease Control, Faculty of Medicine, Oita University, Oita 879-5593, Japan; Research Center for GLOBAL and LOCAL Infectious Diseases, Oita University, Oita 879-5593, Japan

**Keywords:** astrocyte, CCR2, MS, propagermanium, TRAF6

## Abstract

Recent outbreaks of the ZIKA virus (ZIKV) in Brazil and Puerto Rico have been linked to an increase in the incidence of fetal microcephaly and Guillain–Barré syndrome. In addition, although a causal relationship remains unproven, ZIKV has been found in the brains of multiple sclerosis (MS) patients, prompting interest in a possible link. The present study aimed to elucidate the role of ZIKV in the pathogenesis of MS. ZIKV-infected mice with experimental autoimmune encephalomyelitis (EAE) exhibited aggravated EAE symptoms with significant demyelination of the central nervous system (CNS). Moreover, ZIKV infection promoted pathogenic T cell infiltration into the CNS by enhancing the expression of chemokines for C-C motif chemokine receptor 2 (CCR2) in astrocytes, which was dependent on tumor necrosis factor receptor-associated factor 6 (TRAF6) signaling. Propagermanium, a CCR2 inhibitor, prevented ZIKV-induced exacerbation of EAE in mice. These findings highlight the critical role of TRAF6 signaling in the progression of neurological disorders caused by ZIKV infection.

## Teaser

Zika virus infection exacerbates EAE via astrocyte chemokines

## Graphical Abstract

**Graphical Abstract:**
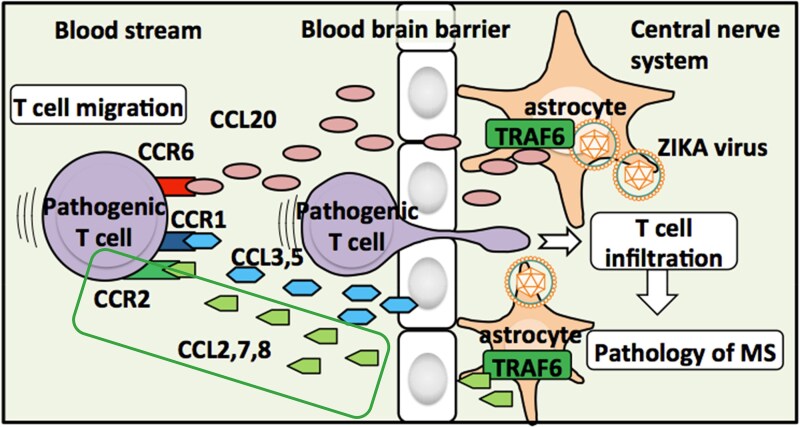

## Introduction

ZIKA virus (ZIKV) is a *Flavivirus* that belongs to the *Flaviviridae* family, which comprises arthropod-borne viruses, such as dengue virus (DENV), yellow fever virus, West Nile virus, and Japanese encephalitis virus. These viruses are widespread globally and are transmitted to humans through the bite of infected mosquito vectors, mainly *Aedes aegypti* and *Aedes albopictus* (1–3). In recent years, there have been several instances of ZIKV outbreaks in Brazil, French Polynesia, and Puerto Rico, which have been correlated with an increase in cases of fetal microcephaly and severe neurological disorders, including Guillain–Barré syndrome (4–8). Multiple sclerosis (MS) is a neurological disorder characterized by autoimmune inflammatory demyelination of the central nervous system (CNS) (9, 10). Although there is no reported association between ZIKV infection and the pathology of MS, recent studies have revealed the presence of ZIKV in the brain tissue of patients with MS (11), indicating that ZIKV infection may play a significant role in the pathogenesis of MS.

Pathogenic T cells, including IL-17-producing helper T cells (Th17 cells), are essential for the development of CNS autoimmunity in patients with MS (12, 13). Mice lacking IL-17 or IL-23, key cytokines required for sustaining Th17 cells (14), are resistant to experimental autoimmune encephalomyelitis (EAE), a widely used animal model of MS (15, 16). Patients with MS exhibit elevated levels of serum IL-17 and IL-23 compared to those of healthy individuals (17). Multiple chemokine/receptor pathways, including Th17 cells that express C-C motif chemokine receptor 2 and 6 (CCR2 and CCR6) (18, 19), are implicated in the pathogenesis of EAE and MS (20–23). Additionally, CCR1-expressing T cells infiltrate the CNS during the development of EAE (24). It has been reported that CCR1, CCR2, and CCR6 are implicated in the pathogenesis of EAE using mouse models deficient for CCR1, CCR2, and CCR6, suggesting a defective migration of leukocyte or T cell into the CNS (18, 25–27). The levels of CCL2, CCL3, and CCL5 are elevated in the blood of patients infected with ZIKV (28, 29 ) and CCL2, CCL3, CCL5, CCL7, and CCL20 are enhanced in the brain of mice infected with ZIKV (30). These findings suggest that ZIKV infection exacerbates both MS and EAE by modulating Th17 cell migration.

Guerrini *et al*. (31) revealed that the receptor activator of nuclear factor-κB (NF-κB) ligand (RANKL), which is expressed on Th17 cells, regulates the production of CCL20 by astrocytes through RANK signaling, leading to the rapid recruitment of pathogenic T cells into the CNS. The tumor necrosis factor receptor-associated factor 6 (TRAF6), which binds to the RANK structural motif, is crucial for RANKL-RANK signal transmission (32, 33). Consequently, TRAF6 signaling in astrocytes has been implicated in the development of EAE. The present study demonstrates that ZIKV infection exacerbates EAE, which is canceled in the absence of TRAF6 in astrocytes. ZIKV infection induces the production of pathogenic T cell-attracting chemokines including CCL2, CCL7, CCL8, and CCL20 in astrocytes in a TRAF6-dependent manner. Furthermore, CCR2-deficient mice and wild-type (WT) mice treated with the CCR2 inhibitor, propagermanium (PG) display resistance to the ZIKV-induced exacerbation of EAE. Thus, the elevation of TRAF6 signaling-dependent CCR2 ligands triggered by ZIKV infection exacerbates EAE because of the migration of pathogenic T cells into the CNS.

## Methods

### Mice

Male and female mice aged 8–12 weeks including C57BL/6, astrocyte-specific TRAF6-deficient mice (TRAF6-floxed mice crossed with GFAP-Cre mice), CCR2-deficient mice, and CCR6-deficient mice, were used in this study. Sex-matched co-housed male and female littermate mice were used for each experiment. C57BL/6 mice were purchased from Japan SLC (Hamamatsu, Japan). TRAF6-floxed and GFAP-Cre mice have been previously described (34, 35). CCR2-deficient and CCR6-deficient mice were generated in a C57BL/6 genetic background using the CRISPR/Cas9 system, as previously reported (36, 37). The mice were maintained in a specific pathogen-free facility at the Division of Laboratory Animal Science at Oita University. All experimental protocols were approved by the Animal Ethics Committee of Oita University (approval numbers: 230901 and 230903).

### Reagents

The MOG35–55 peptide (MEVGWYRSPFSRVVHLYRNGK) was synthesized by SynPeptide (Shanghai, China). Mycobacterium tuberculosis H37RA was purchased from Difco BD Biosciences (Sparks, MD, USA). Complete Freund’s adjuvant (CFA) was procured from BD Biosciences (Franklin Lakes, NJ, USA). Pertussis toxin was obtained from List Biological Labs, Inc. (Campbell, CA, USA). Poly (I:C) was purchased from Sigma-Aldrich (St. Louis, MO, USA). PG was acquired from the Sanwa Kagaku Kenkyusho Company, Ltd. (Aichi, Japan).

### Virus and cells

Zika virus MR 766, PRVABC59, and dengue virus serotype 4 were kindly provided by Rockefeller University (New York, NY, USA). C6/36 cells (*A. albopictus*) were maintained at 28°C in 5% CO_2_ in 10% complete minimum essential medium (MEM) supplemented with Eagle’s MEM (Nissui, Tokyo, Japan), l-alanyl-glutamic acid (Gibco GlutaMAX, Thermo Fisher Scientific, Waltham, MA, USA), non-essential amino acids (Thermo Fisher Scientific), sodium bicarbonate (Thermo Fisher Scientific), and 10% FBS (Hyclone FBS, Thermo Fisher Scientific). The virus was subsequently propagated in C6/36 cells in 2% complete MEM.

### Viral titration

To determine the viral titer, confluent Vero cells were infected with serial dilutions of ZIKV or DENV in 2% complete MEM in 12-well culture plates. Following a 1 h adsorption period at 37°C, 2% methylcellulose (MP Biomedicals, Aurora, OH, USA) was layered on the cells. Subsequently, the cells were incubated for 4 days at 37°C and 5% CO_2_ in a humidified environment, fixed in 10% formalin (Wako, Osaka, Japan), and stained with methylene blue (Wako) to count the plaques.

### Viral infection

Mice were subcutaneously administered 1 × 10^5^  *PFU* of ZIKV or DENV three times, with a 7-day interval, in a volume of 200 μl adjusted with PBS. The control group received an equal volume of 2% complete MEM contained in the viral stock of the experimental group in PBS. EAE was induced 5 days after the last infection.

### Administration of poly (I:C)

Mice were intraperitoneally administered 20 μg of poly (I:C) three times, with a 7-day interval, in a volume of 200 μl adjusted with PBS. The control group received an equal volume of PBS. EAE was induced 5 days after the last administration.

### Induction and assessment of EAE

EAE was induced in mice by subcutaneous immunization with 160 μg MOG35–55 emulsified in CFA containing 400 μg heat-killed *Mycobacterium tuberculosis* H37RA. In addition, the mice received 400 ng of pertussis toxin i.p. on the same day and 2 days later. The mice were assessed for clinical signs of EAE on the following scale: 0, no signs; 1, tail limpness; 2, complete loss of tail tonicity or abnormal gait; 3, partial hind limb paralysis; 4, complete hind limb paralysis; 5, forelimb paralysis; and 6, death.

### Real-time RT-PCR

Total RNA was isolated from the brain, spinal cord, and astrocytes using the TRIzol reagent (Invitrogen, Carlsbad, CA, USA). cDNA was synthesized from RNA (0.5 µg) with the Verso cDNA synthesis kit according to the manufacturer’s instructions (Thermo Scientific, Kanagawa, Japan). Real-time RT-PCR was conducted on a RT-PCR LightCycler96 (Roche, Basel, Switzerland) using a KAPA SYBR FAST qPCR kit (Kapa Biosystems, Inc., Wilmington, MA, USA). All data were normalized to β-actin expression, and the resultant fold-difference relative to β-actin is shown. The amplification conditions were as follows: 45 cycles at 95°C for 5 s and 60°C for 30 s. Primers for *Il17*, *Ifng*, *Csf2*, *Ccl2*, *Ccl3*, *Ccl5*, *Ccl7*, *Ccl8*, *Ccl9*, *Ccl20*, ZIKV, and *β-actin* were purchased from FASMAC. Primer sequences are listed in Supplementary Table 1.

### Isolation of mouse cerebral lymphocytes

Cerebral lymphocytes were obtained from the brain and spinal cord of mice following treatment with collagenase-dispase (Roche) and DNase I (Takara Bio Inc., Shiga, Japan) and subsequently isolated through density gradient centrifugation using Percoll (Sigma-Aldrich) as previously described (38).

### Intracellular cytokine staining and flow cytometry

The intracellular expression of IL-17A and IFN-γ in CD4^+^ T cells was analyzed using fixation/permeabilization concentrate and diluent (eBioscience Inc., San Diego, CA, USA), with Brefeldin A (eBioscience) and Monensin solution (eBioscience), according to the manufacturer’s instructions. Lymphocytes isolated from the brain and spinal cord were incubated with 50 μg/ml phorbol myristate acetate (Sigma-Aldrich), 500 μg/ml calcium ionophore (Sigma-Aldrich), Brefeldin A, and Monensin solution in complete medium at 37°C for 5 h. Following blocking of Fc receptors, surface staining was performed with FITC anti-mouse CD3 (BioLegend, San Diego, CA, USA), PerCP-Cy5.5 anti-mouse CD4 (BioLegend), APC anti-mouse CCR2 (BioLegend), PE-Cy7 anti-mouse CCR6 (BioLegend), and Brilliant Violet 421™ anti-mouse CD3 (BioLegend) for 20 min at 4°C. Intracellular cytokine staining was performed using PE anti-mouse IL-17A, APC anti-mouse IFN-γ, and FITC anti-mouse IFN-γ (all from eBioscience) for 20 min. Dead cells were eliminated using a Zombie Red Fixable Viability Kit (BioLegend). Data were acquired with a FACS BD LSRFortessa X-20 (BD Biosciences) and analyzed using FlowJo software (Tree Star, Inc., Ashland, OR, USA).

### Migration assay

Cultured astrocytes at a density of 4 × 10^5^ cells/well were placed in the lower chamber of a 24-well Transwell plate (Costar, Cambridge, MA, USA, 3 μm pore membrane) and incubated for 24 h. The following day, the astrocytes were infected with ZIKV at an MOI of 0.5 and incubated for an additional 48 h. Subsequently, CD4^+^ T cells were isolated from the spleen of WT mice using MACS technology (Miltenyi Biotec, Tokyo, Japan) and added to the upper wells of Transwell membranes suspended in 200 μl of RPMI 1640 medium. A total of 5 × 10^5^ CD4^+^ T cells were incubated for 5 h at 37°C in a 5% CO_2_ atmosphere. Recombinant CCL20 (PeproTech Inc., Rocky Hill, NJ, USA) was added to the lower chamber (250 ng/ml) as a positive control. The cells on the upper surface of the membrane were removed by washing with PBS. The migrated cells were then fixed and stained with Diff-Quik stain™ (Sysmex, Kobe, Japan). The number of stained T cells was counted in randomly selected non-overlapping fields in the wells using a light microscope.

### Induction of adoptive transfer EAE

Spleens were harvested from mice 11 days after EAE induction, and red blood cells were lysed. Single-cell suspensions were prepared, and splenocytes were cultured in the presence of MOG35–55 (20 μg/ml) and soluble IL-23 (20 ng/ml) for 3 days. After removing the cells attached to the bottom of the dish, they were washed in PBS and injected i.p. into recipient mice (20 million cells/mouse).

### Isolation of astrocytes from mouse brains

Astrocytes were isolated from the brains of three groups of mice: uninfected controls, mice 5 days after the third ZIKV infection, and ZIKV-infected EAE mice 5 days after MOG35–55 immunization, using the Anti-ACSA-2 MicroBead Kit for mice (Miltenyi Biotec). Purified astrocytes were then used for migration assays or intrathecal transfer (2 × 10^5^ cells/mouse, 3 μl PBS at thoracic vertebra 9).

### *In vitro* Th17 differentiation

Naïve CD4^+^ (CD25^−^/CD44^lo^/CD62L^hi^) T cells were isolated from the spleen using magnetic beads (Miltenyi Biotec) and co-cultured with bone marrow-derived dendritic cells at a ratio of 3 × 10^4^ to 9 × 10^4^ in the presence of soluble anti-CD3 (1 μg/ml), anti-CD28 (1 μg/ml), IL-6 (20 ng/ml), TGF-β (2 ng/ml), anti-IFN-γ (10 μg/ml), and anti-IL-4 antibody (10 μg/ml) for a period of 5 days.

### Enzyme-linked immunosorbent assay

Serum samples were obtained from EAE-induced mice on Day 21 following MOG immunization and from mice that were infected with ZIKV three times, with the last infection occurring 5 days prior. MOG (5 μg/ml) and ZIKV (10^5^  *PFU*/ml) were coated overnight in PBS in 96-well Maxisorp plates (Thermo Fisher Scientific). The plates were washed three times, blocked with 1% BSA in PBS containing 0.05% Tween 20 for 1 h, followed by incubation with serum at a dilution of 1/20 for 3 h at room temperature. Following incubation, the plates were washed again and incubated with peroxidase AffiniPure goat anti-mouse IgG (H + L) (Jackson ImmunoResearch, Inc., PA, USA) for 1 h at room temperature. The plates were then washed and incubated with 1 × TMB substrate solution (Invitrogen). Optical density was measured at 450 nm using a Bio-Rad Model 680 Microplate Reader (Bio-Rad Laboratories, Inc., Hercules, CA, USA).

### Statistical analysis

Unpaired Student’s *t*-test was used to evaluate the statistical significance of the variations.

## Results

### ZIKV infection aggravates EAE by enhancing leukocyte infiltration and demyelination in the CNS

**Figure 1.**
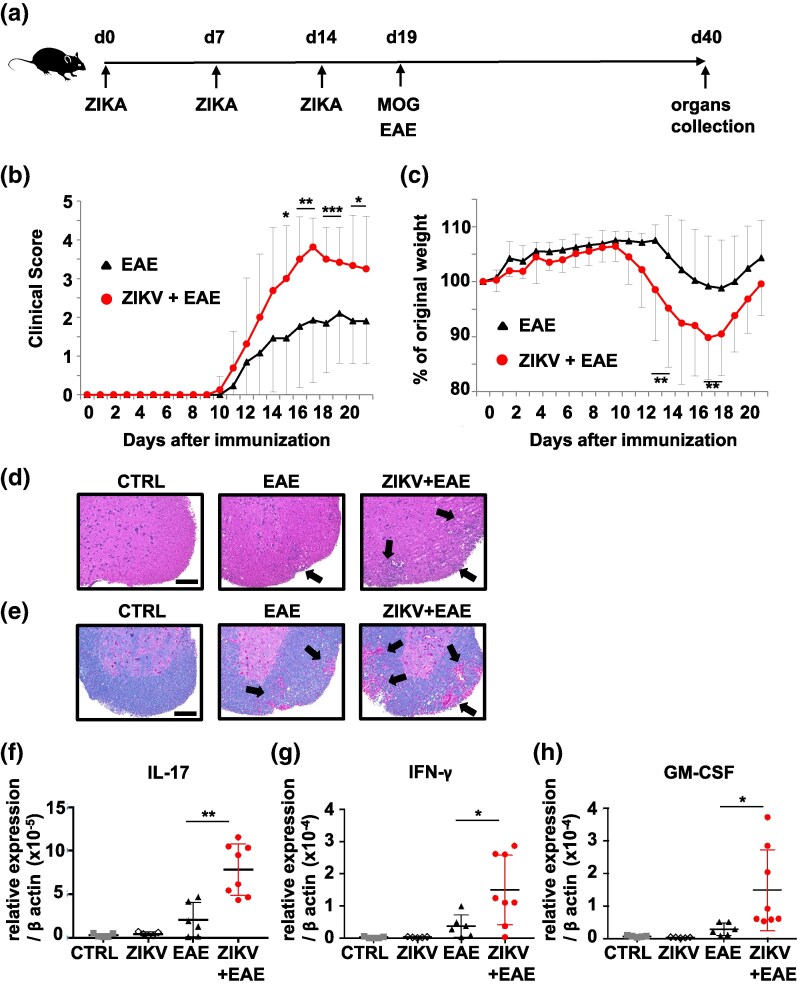
ZIKV infection exacerbates EAE. (a) Schematic illustration demonstrating the process of ZIKV infection and EAE induction by immunization with the MOG35–55/CFA emulsion in WT mice. (b and c) Clinical scores (b) and changes in body weight (c) in EAE (*n* = 17) and ZIKV + EAE mice (*n* = 16) over 21 days following immunization. Data were pooled from four independent experiments. (d and e) Representative hematoxylin and eosin (d) and Luxol fast blue staining (e) of spinal cord sections from CTRL, EAE, and ZIKV + EAE mice obtained on Day 21 postimmunization. Arrows indicate lymphocyte infiltration in (d) and demyelination in (e). Scale bars, 100 μm. (f–h) RT-PCR analysis of *Il17* (f), *Ifng* (g), and *Csf2* (h) in the brain tissue of CTRL, ZIKV, EAE, and ZIKV + EAE mice obtained on Day 21 postimmunization. The results were normalized to those of *β-actin*. CTRL, uninfected mice without EAE; ZIKV, ZIKV-infected mice without EAE. In d–h, data are represented as the mean ± SD of three independent experiments. Unpaired *t*-test; ***, *P* < 0.001; **, *P* < 0.01; *, *P* < 0.05.

To explore the potential role of ZIKV infection in the pathological mechanisms of MS, EAE was induced in WT mice that were repeatedly infected with ZIKV on Days 0, 7, and 14 (Fig. 1a). The control EAE mice were not infected with ZIKV. The mice were subsequently monitored for the clinical onset of symptoms and their severity. The ZIKV + EAE mice exhibited an earlier onset of symptoms at Day 10 accompanied by a higher peak clinical score of 3.81 ± 0.75 on Day 17, as compared to the control EAE mice that showed symptoms on Day 11 and a peak clinical score of 2.10 ± 1.28 on Day 19 (Fig. 1b). Additionally, ZIKV + EAE mice experienced weight loss earlier, beginning on Day 11, and to a greater extent than control mice (Fig. 1c). Notably, ZIKV-infected mice in the absence of EAE (Supplementary Figure 1a) exhibited no clinical symptoms or loss of body weight and were comparable to that of uninfected control (CTRL) mice (Supplementary Figure 1b and c). This suggests that ZIKV infection exacerbates EAE symptoms, but does not trigger them independently.

ZIKV strains are classified into African and Asian types (39). The African strain MR766 was used to infect mice pertaining to the results in Fig. 1b and c. To explore the potential of the Asian strain PRVABC59 to exacerbate EAE, it was used to infect the mice. Although PRVABC59 exacerbated EAE to a certain extent, this effect was less pronounced than that of MR766 (Supplementary Figure 2a and b). Consequently, MR766 was selected for use in subsequent experiments. To assess leukocyte infiltration in the spinal cord 21 days following MOG immunization, hematoxylin and eosin (HE) staining was performed. Enhanced leukocyte infiltration was observed in the spinal cord of EAE mice compared to that of CRTL mice, which was further augmented in ZIKV + EAE mice (Fig. 1d). However, there was no significant leukocyte infiltration in the spinal cord of ZIKV mice without EAE, which is consistent with the findings shown in Supplementary Figures 1b and c (Supplementary Figure 3a). To assess the extent of CNS demyelination, Luxol fast blue (LFB) staining was applied. Patchy loss of myelin was seen in the spinal cord of EAE compared to that in CRTL mice, which was significantly exacerbated in ZIKV + EAE mice (Fig. 1e). Consistent with the HE staining data, no significant demyelination was detected in the CNS of ZIKV mice without EAE (Supplementary Figure 3b). In contrast, infection with DENV did not exacerbate EAE, as demonstrated by the comparable clinical scores and weight loss between the DENV + EAE and EAE groups of mice (Supplementary Figure 4a and b). Additionally, DENV infection did not expedite the accumulation of leukocytes or demyelination in the spinal cord of DENV + EAE mice compared with that in EAE mice (Supplementary Figure 5a and b). Furthermore, we examined whether administration of poly(I:C), a synthetic analog of viral double-stranded RNA and a ligand of Toll-like receptor 3 (TLR3), would exacerbate EAE. However, no increase in clinical EAE scores or body weight loss was observed after poly(I:C) treatment (Supplementary Figure 6). Since no exacerbation of EAE was observed following either DENV infection or poly(I:C) treatment, it is unlikely that TLR3 stimulation alone is sufficient to aggravate EAE pathology. The involvement of Th17 and IL-17^+^ IFN-γ^+^ Th (Th17.1) cells has been implicated in initiating CNS autoimmunity in an EAE model (40). GM-CSF has been identified as a marker for highly pathogenic Th17 cells in MS (41, 42). The mRNA expression of *Il17*, *Ifng*, and *Csf2* was significantly higher in the brain of ZIKV + EAE mice than in those of EAE mice, as detected by real-time RT-PCR (Fig. 1f–h). These results indicate that ZIKV plays a crucial role in the pathogenesis of EAE, which is characterized by the infiltration of pathogenic T cells into the CNS.

### ZIKV infection potentiates the infiltration of pathogenic T cells into the CNS in EAE

**Figure 2.**
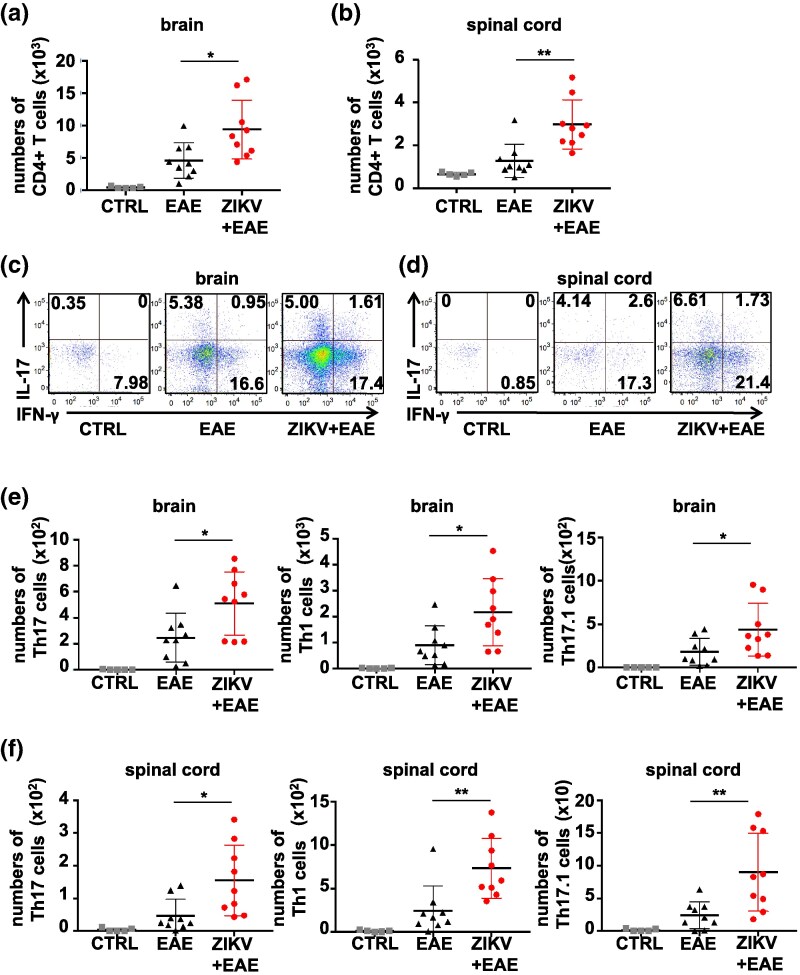
ZIKV infection potentiates T cell infiltration into the CNS in EAE. (a and b) Flow cytometric analysis of lymphocytes, representing the absolute number of CD4^+^ T cells in the brain (a) and spinal cord (b) per mouse, obtained on Day 21 postimmunization with MOG35–55/CFA emulsion in EAE, ZIKV + EAE, and CTRL mice. (c and d) Dot plots gated on CD4^+^ T cells stained intracellularly with anti-IL-17 and anti-IFN-γ antibodies in the brain (c) and spinal cord (d). The numbers represent the percentage of cells in each quadrant. (e and f) Graphs represent the absolute numbers of Th17, Th1, and Th17.1 cells in the brain (e) and spinal cord (f) per mouse. CTRL, uninfected mice without EAE. In a–f, data are presented as the mean ± SD of three independent experiments. Unpaired *t*-test; **, *P* < 0.01; *, *P* < 0.05.

Myelin-specific CD4^+^ T cells play a crucial role in the progression of EAE (43 ). Flow cytometry-based quantification revealed a significantly higher number of CD4^+^ T cells in the brain and spinal cord of ZIKV + EAE mice than that of EAE mice (Fig. 2a and b). Intracellular staining revealed no significant changes in the frequencies of Th17, Th1, and Th17.1 cell subsets between the ZIKV + EAE and EAE groups (Fig. 2c and d); however, the numbers of these subsets in the CNS of ZIKV + EAE mice were significantly greater than those in EAE mice, both in the brain and spinal cord (Fig. 2e and f). In contrast, there was no significant infiltration of Th17, Th1, and Th17.1 cells in the brain and spinal cord of ZIKV mice without EAE (Supplementary Figure 7). These results indicate that ZIKV infection promotes the migration of pathogenic CD4^+^ T cells that produce IL-17, IFN-γ, or both into the CNS of EAE mice.

### ZIKV infection induces the expression of pathogenic T cell-attracting chemokines in astrocytes

**Figure 3.**
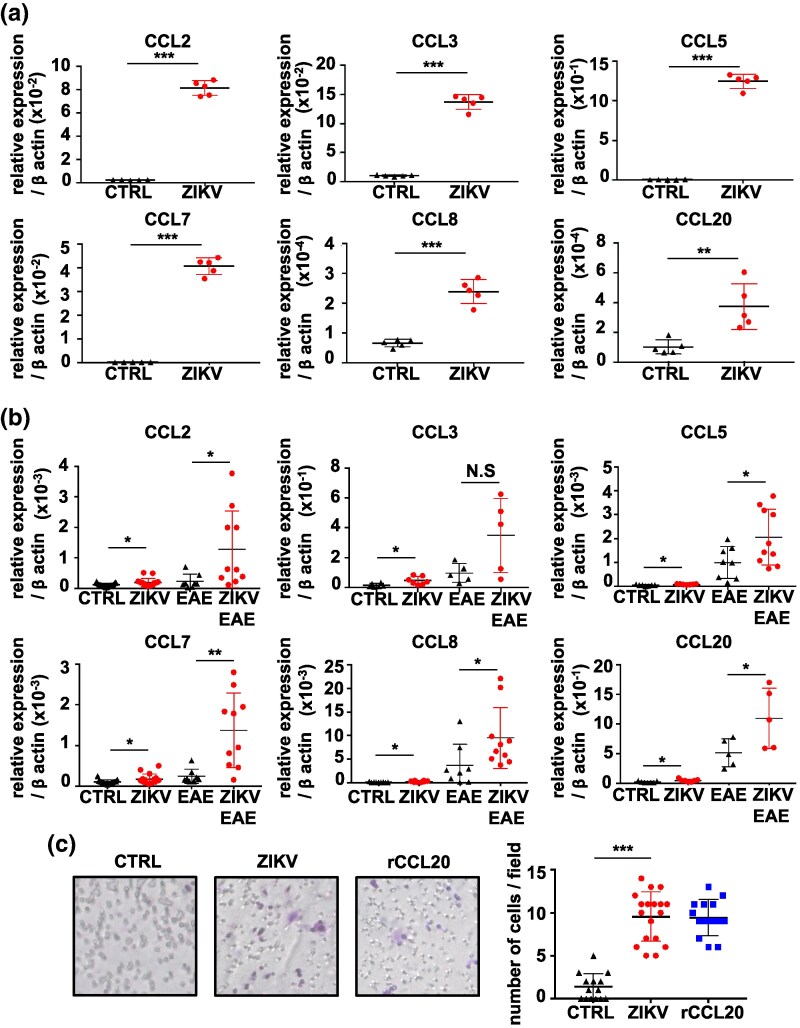
ZIKV infection induces the expression of T cell-attracting chemokines in astrocytes. (a) Quantitative RT-PCR analysis of *Ccl2*, *Ccl3*, *Ccl5*, *Ccl7*, *Ccl8*, and *Ccl20* expression in astrocytes. Cells were infected with ZIKV at an MOI of 0.5, and total RNA was isolated after 2 days. The results were normalized to *β-actin*. (b) Quantitative RT-PCR analysis of *Ccl2*, *Ccl3*, *Ccl5*, *Ccl7*, *Ccl8*, and *Ccl20* expression in the brain tissue of WT mice under four conditions: CTRL, ZIKV, EAE, and ZIKV + EAE. Total RNA was isolated from the brain of ZIKV-infected mice on Day 5 postlast infection, and from EAE and ZIKV + EAE mice on Day 21 postimmunization. The results were normalized to *β-actin*. (c) Transwell chemotaxis assay and quantification of purified CD4^+^ T cells isolated from the spleen using MACS against ZIKV-infected astrocytes with recombinant CCL20 (rCCL20) as the positive control. The number of migrated cells was counted at 5 h postfeeding. The images depict representative Transwell membranes showing migrated cells. The graph represents the number of stained T cells in randomly selected non-overlapping fields of the Transwell membrane. Data are presented as the mean ± SD of three (a) and two (b and c) independent experiments. Unpaired *t*-test; ***, *P* < 0.001; **, *P* < 0.01; *, *P* < 0.05.

Enhanced T cell infiltration in the CNS seen in ZIKV-infected EAE mice led to the hypothesis that these results are from the production of pathogenic T cell-attracting chemokines in the brain and spinal cord. Astrocytes in EAE mice are known to secrete multiple chemokines, which attract Th17 cells into the CNS across the blood-brain barrier (BBB) (31). *In vitro* infection of cultured astrocytes with ZIKV significantly enhanced the mRNA expression of pathogenic T cell-attracting chemokines *Ccl2*, *Ccl3*, *Ccl5*, *Ccl7*, *Ccl8*, and *Ccl20*, but not *Ccl9*, compared with that in the uninfected controls (Fig. 3a and Supplementary Figure 8). Consistent with the astrocyte data, the mRNA expression of *Ccl2*, *Ccl3*, *Ccl5*, *Ccl7*, *Ccl8*, and *Ccl20* was significantly higher in the brain of ZIKV-infected mice than those in uninfected controls. In the EAE-induced group, expression levels of all chemokines except CCL3 were significantly higher in ZIKV-infected mice than in uninfected controls (Fig. 3b). Additionally, ZIKV RNA was detected in the brain following infection (Supplementary Figure 9). In contrast, DENV infection did not alter the expression levels of these chemokines in astrocytes (Supplementary Figure 10) or the brain (Supplementary Figure 11).

T cell migration in response to ZIKV infection in astrocytes was analyzed using a Transwell assay with CD4^+^ T cells isolated from the spleen by magnetic-activated cell sorting (MACS). The migration of CD4^+^ T cells was significantly higher in ZIKV-infected astrocytes than that of the uninfected controls (Fig. 3c). These results suggest that ZIKV-infected astrocytes produce substantial levels of T cell-attracting chemokines that promote the migration of pathogenic T cells into the CNS through the chemokine/receptor axis in EAE mice.

To seek other possible mechanisms of exacerbation of EAE, we immunized WT mice with ZIKV particles along with an adjuvant. We found neither EAE induction nor anti-MOG antibody production in the mice (Supplementary Figure 12a–c), suggesting that the exacerbation of EAE by ZIKV infection is not attributed to cross-reaction between anti-ZIKV antibodies and myelin protein on nerve axons. Moreover, the presence of ZIKV did not affect Th17 cell differentiation of naïve T cells cultured under Th17 differentiation conditions (Supplementary Figure 12d). Therefore, the exacerbation of EAE by ZIKV infection is caused by an enhanced migration of T cells toward the CNS.

### Astrocyte-specific TRAF6-deficient mice are resistant to ZIKV-induced exacerbation of EAE

**Figure 4.**
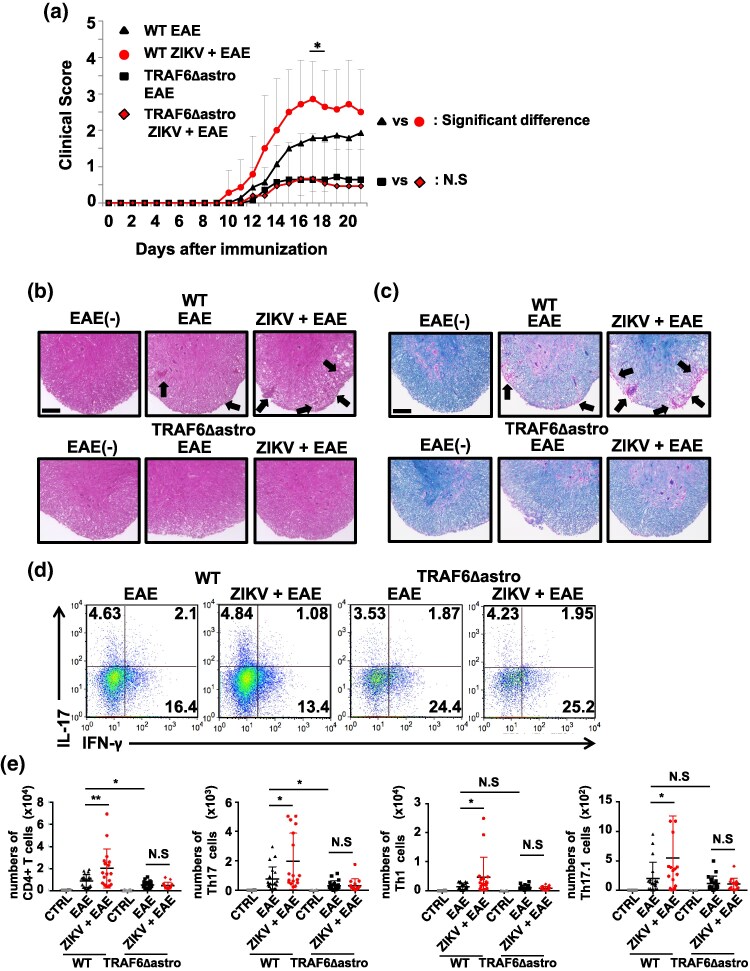
Astrocyte-specific TRAF6-deficient mice are protected against ZIKV-induced exacerbation of EAE. (a) Clinical scores of WT EAE (*n* = 14), WT ZIKV + EAE (*n* = 14), TRAF6Δastro (*n* = 14), and TRAF6Δastro ZIKV + EAE mice (*n* = 15) over 21 days following immunization with MOG35–55/CFA emulsion. (b and c) Representative hematoxylin and eosin (b) and Luxol fast blue staining (c) of spinal cord sections of mice treated as described in (a). Arrows indicate lymphocyte infiltration in (b) and demyelination in (c). Scale bar, 100 μm. (d) Flow cytometric analysis of lymphocytes representing the absolute number of CD4^+^ T cells in the brain of mice treated as in (a). Dot plots gated on CD4^+^ T cells stained intracellularly with anti-IL-17 and anti-IFN-γ antibodies. The number represents the percentage of cells in each quadrant. (e) Graphs represent the absolute numbers of CD4^+^ T, Th17, Th1, and Th17.1 cells in the brain. CTRL, uninfected mice without EAE. Data are pooled from two independent experiments in (a). Data are representative of two independent experiments in (b–e). Graphs represent mean ± SD. Unpaired *t*-test; **, *P* < 0.01; *, *P* < 0.05; N.S., not significant.

The RANK-RANKL signaling pathway mediated by TRAF6 in astrocytes is crucial for chemokine production and Th17 cell migration into the CNS in EAE mice (31–33). Hence, it was hypothesized that astrocyte-specific TRAF6-deficient (TRAF6Δastro) mice would be resistant to ZIKV-induced EAE exacerbation. The exacerbation of EAE induced by ZIKV was diminished in TRAF6Δastro mice, suggesting critical involvement of TRAF6-signaling in astrocytes (Fig. 4a). HE and LFB staining revealed reduced leukocyte infiltration and demyelination, respectively, in the spinal cord sections of TRAF6Δastro mice compared to those in WT mice (Fig. 4b and c). Flow cytometry-based quantification revealed no significant changes in the frequencies of Th17 and Th17.1 cell subsets in the brain of ZIKV-infected WT and TRAF6Δastro mice (Fig. 4d). However, unlike in WT mice, an increase in the number of CD4^+^ T cells including Th17, Th1 and Th17.1 cells was severely suppressed in TRAF6Δastro mice with EAE and further enhancement of T cell numbers by ZIKV infection was completely abolished (Fig. 4e). These findings suggest that TRAF6 signaling in astrocytes is essential for the enhanced migration of CD4^+^ T cells into the CNS during ZIKV infection.

### ZIKV promotes the chemokine expressions for CCR2 and CCR6, but not for CCR1 in astrocytes in a TRAF6-dependent manner

**Figure 5.**
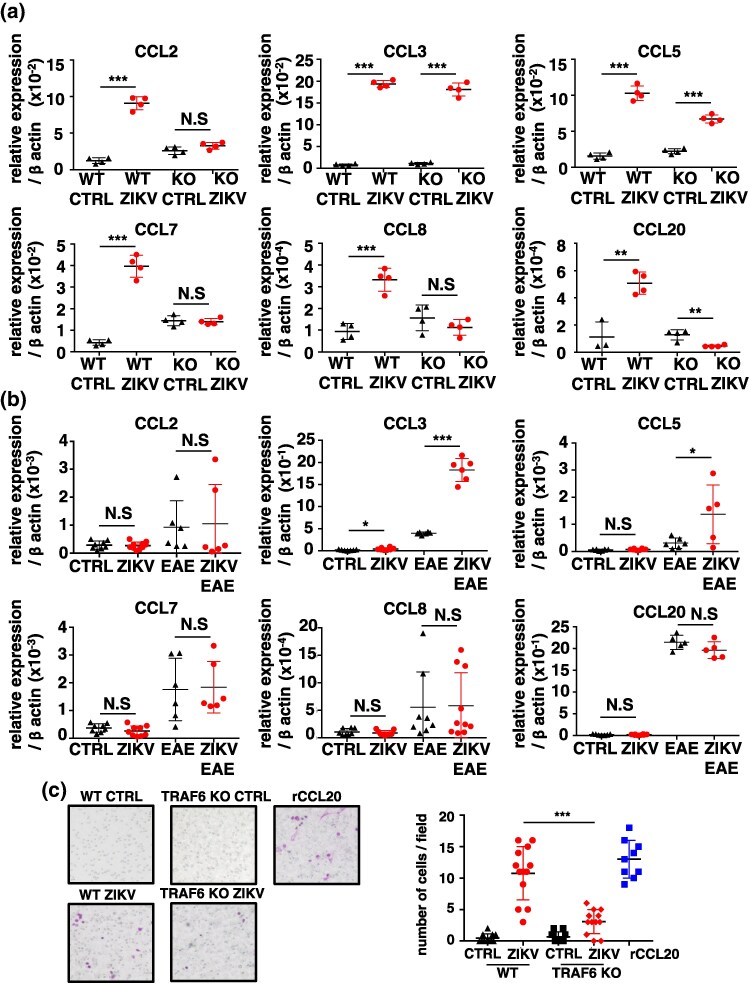

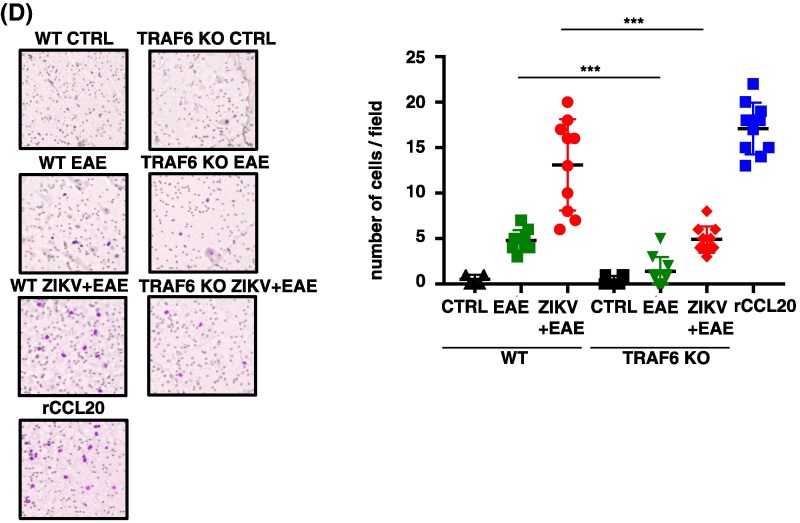
ZIKV promotes TRAF6-dependent induction of CCR2 and CCR6, but not CCR1 chemokines in astrocytes. (a) Quantitative RT-PCR analysis of *Ccl2*, *Ccl3*, *Ccl5*, *Ccl7*, *Ccl8*, and *Ccl20* expression in WT and TRAF6-deficient astrocytes. Cells were infected with ZIKV at an MOI of 0.5, and total RNA was isolated after 2 days. The results were normalized to *β-actin*. (b) Quantitative RT-PCR analysis of *Ccl2*, *Ccl3*, *Ccl5*, *Ccl7*, *Ccl8*, and *Ccl20* in the brain tissue of TRAF6Δastro mice under four conditions: CTRL, ZIKV, EAE, and ZIKV + EAE. Total RNA of ZIKV TRAF6Δastro mice was isolated from the brain on Day 5, postlast infection, or total RNA of EAE and ZIKV + EAE TRAF6Δastro mice was isolated on Day 21 postimmunization. The results were normalized to *β-actin*. (c, d) Transwell chemotaxis assay and quantification of purified CD4^+^ T cells isolated from the spleen using MACS against ZIKV-infected WT and TRAF6-deficient astrocytes (c) or astrocytes isolated from brains of CTRL, ZIKV, EAE, and ZIKV + EAE WT and TRAF6Δastro mice (d), with recombinant CCL20 (rCCL20) as the positive control. The number of migrated cells was counted 5 h postfeeding. The images depict representative Transwell membranes showing migrated cells. The graph represents the number of stained T cells in randomly selected non-overlapping fields of the Transwell membrane. Data are presented as the mean ± SD of three (a) and two (b–d) independent experiments. Graphs represent mean and SD values. Unpaired *t*-test; ***, *P* < 0.001; **, *P* < 0.01; *, *P* < 0.05; N.S., not significant.

To explore the mechanism by which TRAF6 deficiency leads to the impaired migration of CD4^+^ T cells into the CNS, we infected TRAF6-deficient astrocytes with ZIKV *in vitro* and analyzed chemokine expression levels. In the uninfected state, the expression levels of *Ccl2*, *Ccl7*, and *Ccl8* tended to be slightly higher in TRAF6-deficient astrocytes than in WT astrocytes. The induction of *Ccl2*, *Ccl7*, *Ccl8*, and *Ccl20* mRNA by ZIKV infection observed in WT astrocytes was completely abolished in TRAF6-deficient astrocytes (Fig. 5a). On the other hand, *Ccl3* and *Ccl5* mRNA levels were similarly induced irrespective of TRAF6 expression. Consistent with these results, the mRNA expression of *Ccl3*, but not *Ccl2*, *Ccl5*, *Ccl7*, *Ccl8*, and *Ccl20,* was enhanced in the brains of ZIKV-infected TRAF6Δastro mice compared with those in uninfected controls (Fig. 5b). Furthermore, when comparing the EAE and ZIKV + EAE groups, *Ccl3* and *Ccl5* expression levels were further increased in the ZIKV + EAE group, whereas no such enhancement was observed for *Ccl2*, *Ccl7*, *Ccl8*, or *Ccl20*. These results indicate that CCR2 ligands CCL2, 7, and 8, and CCR6 ligand CCL20, but not CCR1 ligands CCL3 and 5, highly depend on TRAF6 signaling pathway.

Transwell migration assays revealed that the migration of CD4^+^ T cells toward ZIKV-infected TRAF6-deficient astrocytes was significantly lower than that toward ZIKV-infected WT astrocytes (Fig. 5c). We also isolated astrocytes from the brains of WT and TRAF6Δastro mice under ZIKV-infected or ZIKV + EAE conditions and performed T-cell migration assays. Consistent with the results obtained from cultured astrocytes shown in Fig. 5c, WT ZIKV-infected astrocytes were significantly more effective at attracting T cells than TRAF6-deficient astrocytes (Fig. 5d). Furthermore, although WT ZIKV-infected astrocytes exhibited markedly enhanced T-cell attraction under EAE conditions, this effect was significantly reduced in TRAF6-deficient astrocytes.

These results suggest that TRAF6 signaling in astrocytes regulates the expression of chemokine ligands for CCR2 and CCR6 to promote pathogenic T cell infiltration into the CNS during ZIKV infection.

### ZIKV does not alter the expression of chemokine receptors on T cells

The effect of ZIKV infection on the expression of chemokine receptors on T cells was further analyzed. Accordingly, the expression of CCR2 and CCR6 on CD4^+^ T, Th17, Th1, and Th17.1 cells was analyzed in the brain and spleen of EAE-induced mice. In both WT and TRAF6Δastro mice, most of CD4^+^ T, Th17, Th1, and Th17.1 cells in the brain were doubly positive for CCR2 and CCR6, and most of them in the spleen were intermediately positive for CCR2 and CCR6 (Supplementary Figure 13a). The frequencies of these T cell subsets were unchanged by ZIKV infection (Supplementary Figure 13a). Moreover, the geometric mean fluorescence intensity of CCR2 and CCR6 on T cells was not altered by ZIKV infection (Supplementary Figure 13b). These results indicate that ZIKV infection does not influence the expression of chemokine receptors on T cells.

### Zika virus infection does not impact T cells directly, but rather affects the environment surrounding T cells

**Figure 6.**
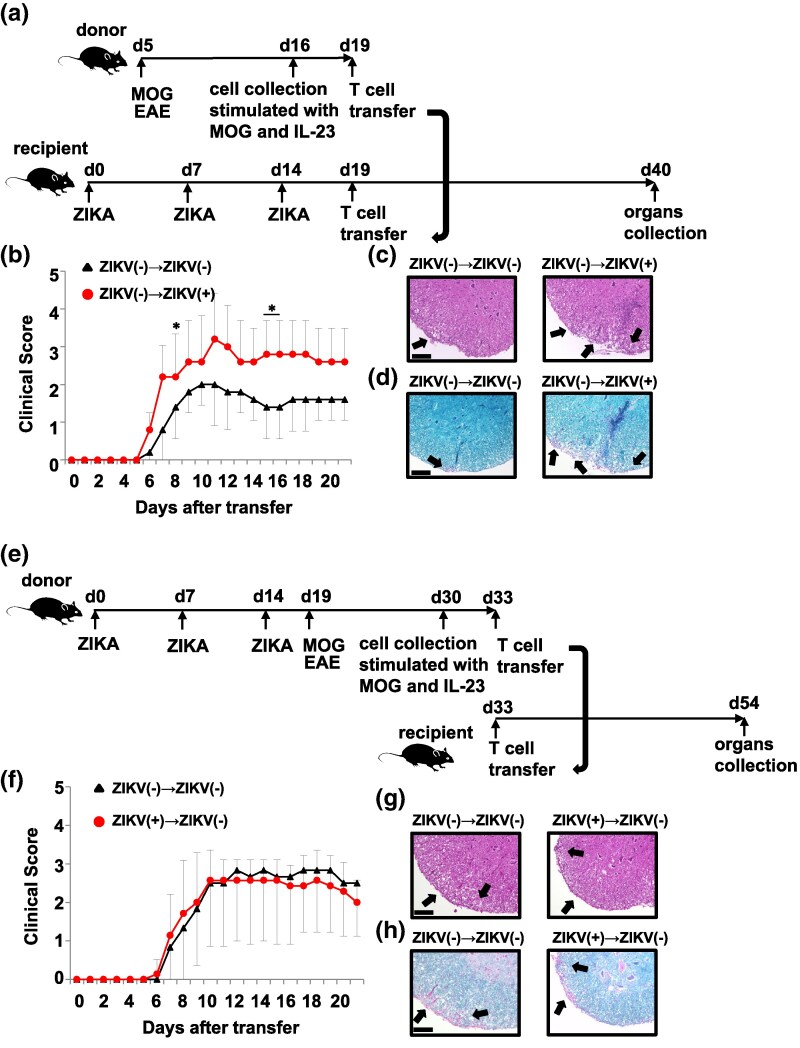

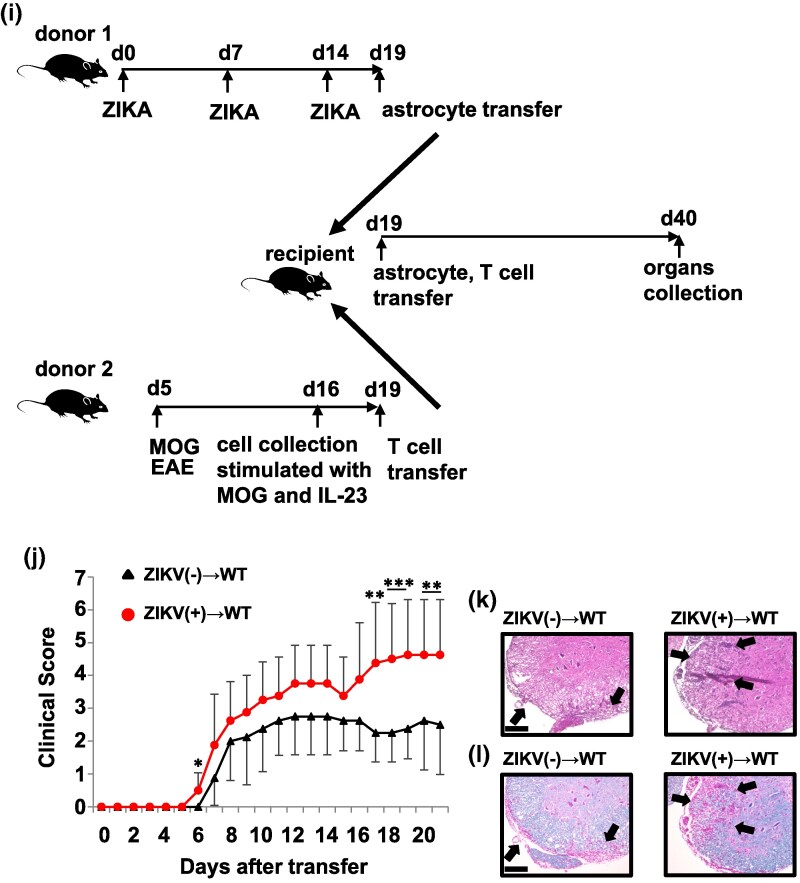
ZIKV does not alter the expression of chemokine receptors on T cells. (a) Schematic illustration of adoptively transferred EAE model. (b) Clinical scores of uninfected WT recipient mice transferred with T cells from uninfected WT (*n* = 5) and ZIKV-infected WT recipient mice transferred with T cells from uninfected WT mice (*n* = 5) over a period of 21 days following the transfer of T cells. (c and d) Representative hematoxylin and eosin (c) and Luxol fast blue staining (d) of spinal cord sections of mice as treated in (a). The arrows indicate lymphocyte infiltration in (c) and demyelination in (d). Scale bars, 100 μm. (e) Schematic illustration of the adoptively transferred EAE model. (f) Clinical scores of uninfected WT recipient mice transferred with T cells from uninfected WT (*n* = 6) and uninfected WT recipient mice transferred with T cells from ZIKV-infected WT mice (*n* = 7) over a period of 21 days following the transfer of T cells. (g and h) Representative hematoxylin and eosin (g) and Luxol fast blue staining (h) of spinal cord sections of treated mice, as in (e). Arrows indicate lymphocyte infiltration in (g) and demyelination in (h). Scale bars, 100 μm. (i) Schematic illustration of the adoptively transferred EAE model. (j) Clinical scores of WT recipient mice transferred with astrocytes from uninfected WT (*n* = 8) and ZIKV-infected WT recipient mice (*n* = 8) over a period of 21 days following the transfer of T cells. (k and l) Representative hematoxylin and eosin (k) and Luxol fast blue staining (l) of spinal cord sections of treated mice, as in (i). Arrows indicate lymphocyte infiltration in (k) and demyelination in (l). Scale bars, 100 μm. Data are representative of two independent experiments in (c, d, g, h, k, and l) and pooled from two independent experiments in (b, f, and j). Graphs represent the mean and SD values. Unpaired *t*-test; ***, *P* < 0.001; **, *P* < 0.01; *, *P* < 0.05.

To ensure the direct impact of ZIKV on astrocytes through the modulation of chemokine expression, splenic T cells collected from EAE-induced mice without ZIKV infection were adoptively transferred into recipient mice either with or without ZIKV infection (Fig. 6a). The ZIKV-infected recipient mice exhibited an average clinical score of 3.0 on Day 11, as compared to the uninfected recipient mice that remained below 2.0 throughout the experimental period (Fig. 6b). HE and LFB staining revealed enhanced leukocyte infiltration and severe demyelination in ZIKV-infected recipient mice compared to that in uninfected recipient mice (Fig. 6c and d).

In contrast, when splenic T cells from uninfected or ZIKV-infected mice with EAE were adoptively transferred to uninfected recipient mice (Fig. 6e), the mean clinical scores, leukocyte infiltration, and CNS demyelination were comparable between the two groups (Fig. 6f, g, and h, respectively). These results suggest that ZIKV promotes T cell migration by modulating the microenvironment surrounding T cells.

Furthermore, to determine whether astrocytes contribute to the inflammatory microenvironment, astrocytes were isolated from ZIKV-infected mice and transferred into uninfected recipient mice. In parallel, splenic T cells from uninfected EAE donor mice were adoptively transferred, thus establishing an adoptive transfer EAE model (Fig. 6i). Mice that received astrocytes from ZIKV-infected donors exhibited significantly higher clinical scores than those that received astrocytes from uninfected donors (Fig. 6j). HE and LFB staining further demonstrated that the transfer of ZIKV-infected astrocytes exacerbated leukocyte infiltration and demyelination (Fig. 6k and l). These data indicate that ZIKV-infected astrocytes modulate T cell migration in mice with EAE.

### ZIKV-mediated exacerbation of EAE is dependent on CCR2 but not CCR6

**Figure 7.**
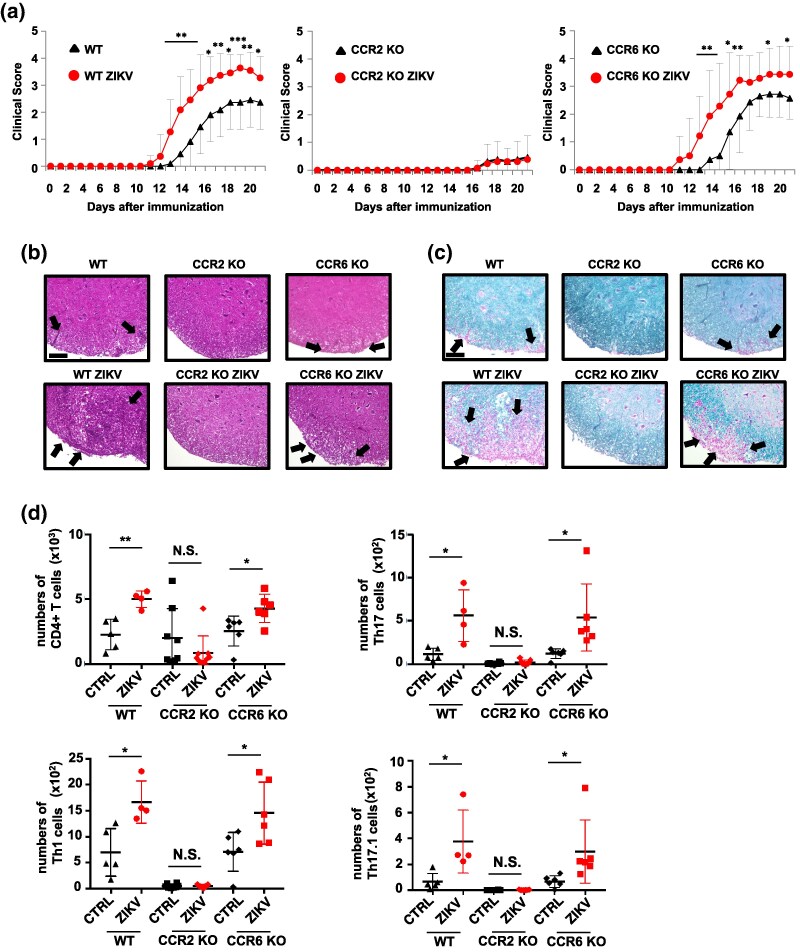
ZIKV-induced exacerbation of EAE is abolished in CCR2-deficient mice (a) clinical scores of uninfected WT (*n* = 11), ZIKV-infected WT (*n* = 11), uninfected CCR2-deficient (*n* = 13), ZIKV-infected CCR2-deficient (*n* = 13), uninfected CCR6-deficient (*n* = 14), and ZIKV-infected CCR6-deficient mice (*n* = 14) over 21 days following immunization with MOG35–55/CFA emulsion. (b and c) Representative hematoxylin and eosin (b) and Luxol fast blue staining (c) of spinal cord sections from treated mice as described in (a). The arrows indicate lymphocyte infiltration in (b) and demyelination in (c). Scale bars, 100 μm. (d) Flow cytometric analysis of lymphocytes representing the absolute number of CD4^+^ T cells in the brain obtained on Day 21 postimmunization with the MOG35–55/CFA emulsion. CD4^+^ T cells were intracellularly stained with anti-IL-17 and anti-IFN-γ antibodies. The numbers of CD4^+^ T, Th17, Th1, and Th17.1 cells in the brain are represented. Data were pooled from two independent experiments in (a). Data are representative of two independent experiments in (b–d). The graphs represent mean and SD values. Unpaired *t*-test; ***, *P* < 0.001; **, *P* < 0.01; *, *P* < 0.05; N.S., not significant.

To determine the critical chemokine/receptor axis associated with the ZIKV-induced exacerbation of EAE, EAE was induced in CCR2- or CCR6-deficient mice with ZIKV infection. ZIKV infection failed to exacerbate EAE in CCR2-deficient mice judged by clinical scores; however, this was not the case for CCR6-deficient mice (Fig. 7a). HE and LFB staining revealed significant leukocyte infiltration and demyelination in the spinal cord of WT and CCR6-deficient mice following ZIKV infection, but not in CCR2-deficient mice (Fig. 7b and c). Flow cytometry-based quantification revealed significantly increased CD4^+^ T, Th17, Th1, and Th17.1 cells in the brain of WT and CCR6-deficient mice following ZIKV infection; however, the numbers of those T cell subsets were apparently low in the absence of CCR2 even in uninfected EAE mice (Fig. 7d). These results indicate that the CCR2 ligands/CCR2 axis is essential for the development of EAE as well as rapid migration of pathogenic CD4^+^ T cells into the CNS.

### PG suppresses the exacerbation of EAE caused by ZIKV infection

**Figure 8.**
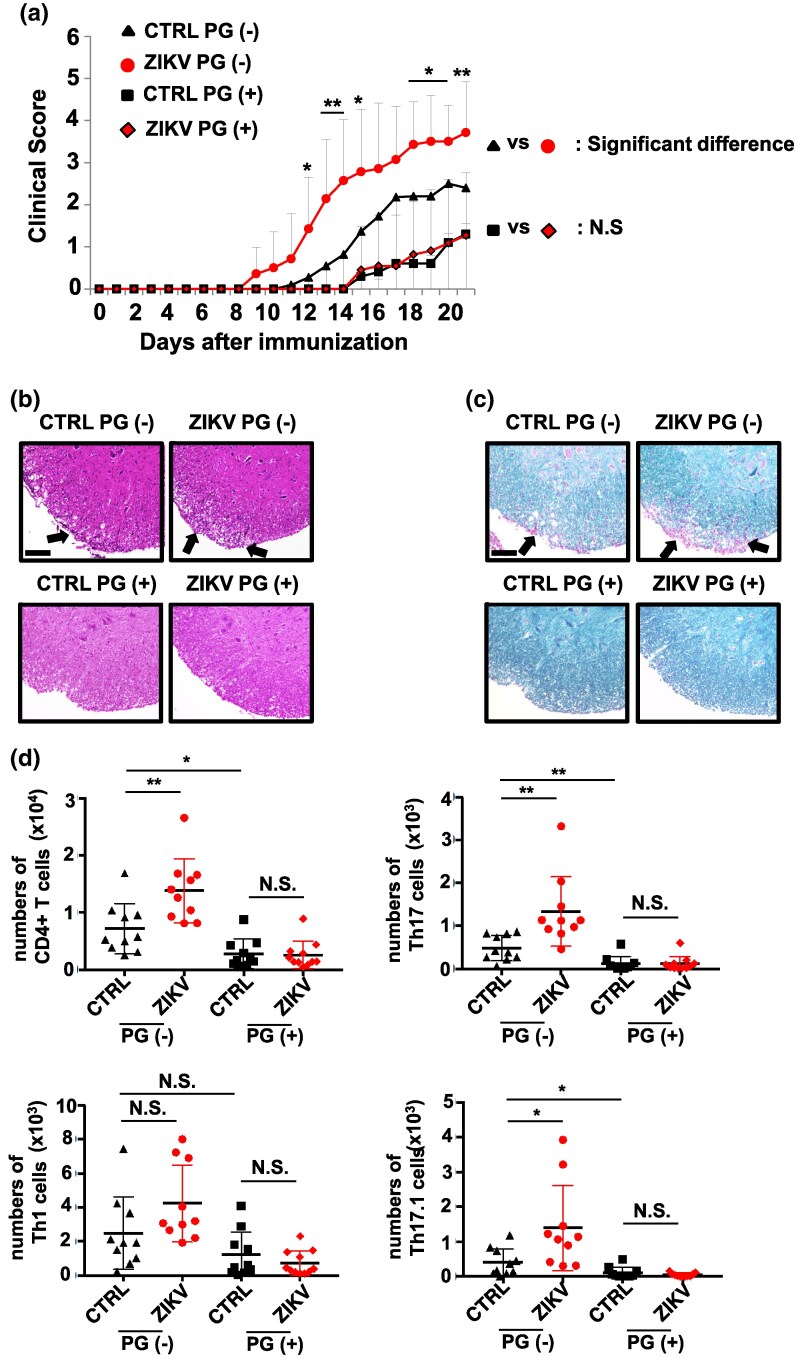
Propagermanium efficiently suppresses the exacerbation of EAE by ZIKV infection. (a) Clinical scores of uninfected control (*n* = 11), ZIKV-infected control (*n* = 14), uninfected PG-administered (*n* = 10), and ZIKV-infected PG-administered mice (*n* = 11) over a period of 21 days following immunization with MOG35–55/CFA emulsion. Starting the day prior to MOG immunization, a daily oral dose of 5 mg/kg PG in 200 μl PBS was administered. The control group received 200 μl of PBS as the vehicle. (b and c) Representative hematoxylin and eosin (b) and Luxol fast blue staining (c) of spinal cord sections from treated mice, as in (a). Arrows indicate lymphocyte infiltration in (b) and demyelination in (c). Scale bars, 100 μm. (d) Flow cytometric analysis of lymphocytes, representing the absolute number of CD4^+^ T cells in the brain of mice as treated in (a). CD4^+^ T cells were stained intracellularly with anti-IL-17 and anti-IFN-γ antibodies. The numbers of CD4^+^ T, Th17, Th1, and Th17.1 cells in the brain are shown. CTRL, uninfected mice with EAE. PG (+), PG-administered mice. Data were pooled from two independent experiments in (a) and two independent experiments in (b–d). The graphs represent mean and SD values. Unpaired *t*-test; **, *P* < 0.01; *, *P* < 0.05; N.S., not significant.

The efficacy of the oral administration of the CCR2 inhibitor PG on ZIKV infection-mediated exacerbation of EAE was further analyzed. As shown in Fig. 1, EAE was exacerbated by ZIKV infection in vehicle-treated group (Fig. 7a). Interestingly, however, EAE was attenuated in PG-treated group, and ZIKV-induced exacerbation of EAE was completely diminished by PG administration (Fig. 8a). HE and LFB staining revealed no significant ZIKV-mediated leukocyte infiltration or demyelination in the spinal cord of PG-treated mice, whereas the vehicle-treated group exhibited marked pathological changes (Fig. 8b and c). Consistent with Fig. 8a, uninfected PG-treated mice had significantly lower CD4^+^ T, Th17, and Th17.1 cells than that of uninfected control mice. Importantly, PG treatment completely inhibited the ZIKV-induced increase in each T cell subset in the brain (Fig. 8d). These results indicate that PG treatment suppresses the EAE regardless of ZIKV infection as observed in CCR2-deficient mice.

## Discussion

The present study identified the mechanistic role of the CCR2 ligands/CCR2 axis in enhancing the migration of pathogenic T cells into the CNS, leading to the exacerbation of EAE in ZIKV infection. These results support the possibility that ZIKV infection aggravates MS in humans. Although ZIKV infection alone did not induce EAE symptoms (Supplementary Figure 1a), the direct association of ZIKV infection with the onset of MS remains uncertain. Nevertheless, it is conceivable that the confluence of ZIKV infection and other contributing factors may be instrumental in the pathogenesis of MS. Further research is necessary to explore this possibility. The lack of vaccine and therapeutic medication for ZIKV poses a threat of increased severe MS cases during a new ZIKV pandemic. Therefore, it is crucial to investigate the potential impact of ZIKV infection on MS and prevention strategies.

Drugs that inhibit pathogenic T cell migration are potential therapeutic options for MS. For instance, fingolimod inhibits the egress of T cells from lymph nodes by targeting sphingosine-phosphate receptors (44), while natalizumab blocks T cell migration by binding to the α4 subunit of integrin, and preventing the interaction between α4β1 integrin and VCAM-1 (45). Currently, there are no specific drugs available for targeting chemokines or chemokine receptors for the treatment of MS. Chemokines facilitate the migration of pathogenic T cells into the CNS during EAE development. Astrocytes, which produce chemokines, regulate the migration of these cells and inflammation in the CNS. For example, astrocyte-specific CCL2-deficient mice were resistant to EAE (46). Thus, astrocyte-derived chemokines play crucial roles in EAE development. In the present study, ZIKV infection led to a significant increase in the expression of pathogenic T cell-attractant chemokines, such as CCL2 in astrocytes (Fig. 3a). Conversely, this induction was not detected in astrocytes infected with DENV, a virus belonging to the same virus family (Flaviviridae) as ZIKV and sharing highly conserved amino acid sequences (Supplementary Figure 9) (47). Thus, future studies are warranted to elucidate the mechanism by which ZIKV, but not DENV, potently stimulates chemokine production in the CNS and to explore the possibility of ZIKV as a causative agent for various neurological disorders, such as Guillain–Barré syndrome.

We also aimed to explore whether novel factors beyond elevated chemokines in the CNS could aggravate EAE during ZIKV infection. Patients infected with ZIKV produce antibodies that cross-react with MOG peptides (48), implying that ZIKV may trigger an adaptive immune response against MOG peptides and self-myelin epitopes in the CNS. This may be due to certain epitopes in the ZIKV genome that cross-react with the MOG. In this study, MOG-specific IgG was not detected in ZIKV-infected mice (Supplementary Figure 11b). Additionally, the replacement of MOG peptides with ZIKV did not elicit EAE-like symptoms in the EAE models (Supplementary Figure 11a). Naïve CD4^+^ T cells infected with HCV under Th17 cell-polarizing conditions significantly enhanced Th17 cell differentiation (49). In the present study, ZIKV infection of cultured naïve CD4^+^ T cells did not promote Th17 cell differentiation (Supplementary Figure 11d). The direct effect of ZIKV on the expression of chemokine receptors, which promote T cell infiltration into the CNS, was analyzed. The expression of CCR2 and CCR6 in each of the T cell subsets was not altered in the ZIKV-infected mice with EAE (Supplementary Figure 12a and b). In the transfer EAE model, ZIKV-infected recipient mice experienced significant exacerbation of EAE symptoms, unlike recipient mice that received T cells from ZIKV-infected donor mice (Fig. 6b and f). These results indicate that the ZIKV-mediated exacerbation of EAE is solely due to an increase in the expression of T cell-attracting chemokines from astrocytes, rather than alternate factors.

Because WT mice are resistant to ZIKV infection, IFNAR1- or STAT1-deficient mice are used as models for lethal ZIKV infection (50 , 51). In this study, no significant behavioral changes or weight loss were observed in ZIKV-infected WT mice (Supplementary Figure 1b and c). However, following ZIKV infection, T cell-attracting chemokines were induced in the CNS of the WT mice (Fig. 3b). Higher levels of ZIKV RNA were detected in the CNS of infected C57BL/6 mice at 6 days than at 2 days postinfection (50). In the present study, ZIKV RNA was detected in the brain of WT mice, together with the induction of cerebral chemokines, following three repeated infections (Supplementary Figure 8), rather than after a single infection (data not shown). Thus, WT mice have a robust immune response that rapidly eliminates ZIKV from astrocytes, followed by a transient inflammatory reaction. ZIKV infection alone did not trigger T cell infiltration (Supplementary Figure 6) or demyelination (Supplementary Figure 3b) in the CNS. Although some of the IFNAR1-deficient and STAT1-deficient mice showed EAE-like symptoms at an early stage of ZIKV infection, there was no infiltration of immune cells in the CNS (data not shown). Therefore, ZIKV is thought to directly infect the CNS, causing behavioral abnormalities in those ZIKV infection models. According to these findings, the development of MS in humans may result from a combination of ZIKV infection and other potential contributing factors.

In astrocytes, NF-κB-dependent pathways trigger the induction of chemokines CCL2, CCL20, CCL3, and CCL5 (52–56). Our previous study revealed that pro-inflammatory molecules are induced by TRAF6-mediated activation of NF-κB and MAP kinases in response to TLR ligands in dendritic cells (57). Accordingly, it could be speculated that activation of the NF-κB pathway in astrocytes by ZIKV infection could lead to the production of chemokines. T cell-specific RANKL-deficient mice are resistant to EAE, attributed to RANKL-RANK signaling-dependent inhibition of chemokine production in astrocytes (31). Since TRAF6 is crucial for RANKL-RANK signal transmission (32, 33), it was hypothesized that the expression of several chemokines would be inhibited in TRAF6-deficient astrocytes. ZIKV infection-dependent elevation of CCL2, CCL7, CCL8, and CCL20, but not of CCL3 and CCL5, was inhibited in TRAF6-deficient astrocytes (Fig. 5a), indicating that ZIKV induced the expression of CCR2 and CCR6 ligands, but not CCR1 ligands, in a TRAF6-dependent manner. Interestingly, ZIKV infection did not upregulate CCL9, a CCR1 ligand (Supplementary Figure 7). TLRs and RIG-I-like receptors are pattern recognition receptors that recognize viral RNA. Both TLR/NF-κB and RIG-I/NF-κB signaling pathways are partially mediated by TRAF6 (58, 59), and ZIKV may additionally activate other signaling pathways, independent of TRAF6, to produce chemokines such as CCL3 and CCL5.

Genetically, ZIKV strains are classified as either African or Asian (39), with all large-scale epidemic-causing strains being classified as Asian. African strains have not been directly linked to neurological disorders, suggesting that Asian strains may be more capable of causing neurodegenerative diseases than African strains (60, 61). However, it has been reported that the effectiveness of African strains has high transmissibility and fetal pathogenicity in mice, and greater transmission efficiency to mosquito vectors when compared to that of Asian strains (62, 63). In the present study, the exacerbation of EAE was more pronounced in mice infected with the African strain than in those infected with the Asian strain (Supplementary Figure 2a and b), implying that African strains also play a pivotal role in the development of neurological disorders.

The present study highlights the mechanistic insight of ZIKV in aggravating EAE and implicates its role as a contributing factor to the progression of MS. Further studies are warranted to decipher the association between ZIKV infection history and incidence of MS. EAE exacerbation is attributed to ZIKV-induced TRAF6 signaling-mediated production of chemokines associated with CCR2 in astrocytes. Therapeutic drugs that inhibit CCR2 or TRAF6 signaling could potentially prevent the exacerbation of neurological disorders triggered by ZIKV infection. The CCR2 inhibitor PG, a therapeutic drug for cancer and liver injury (64–66), effectively prevented the ZIKV-induced exacerbation of EAE and significantly reduced the clinical score in uninfected mice (Fig. 8a). In this study, PG was administered orally at a daily dose beginning one day prior to the induction of EAE. Although PG treatment significantly reduced the severity of EAE, some PG-treated mice had severe EAE outcomes. This suggests that the protective effect of PG may depend on the stage of EAE, and that PG may be effective at specific phases of remission and relapse in MS patients. With these clarifications, PG may be a therapeutic option for MS in terms of drug repositioning.

## Supplementary Material

dxaf075_Supplementary_Data
