# Supplementary material for: Zika virus exacerbates encephalomyelitis by inducing the production of T cell-attracting chemokines in astrocytes
Source: Int Immunol. 2025 Dec 17;38(5):318–34. doi: 10.1093/intimm/dxaf075 (PMC13150445; doi:10.1093/intimm/dxaf075)
Supplement: dxaf075_Supplementary_Data [file dxaf075_supplementary_data.zip › Figure_International immunology FigureS1.pptx]

## Slide 1
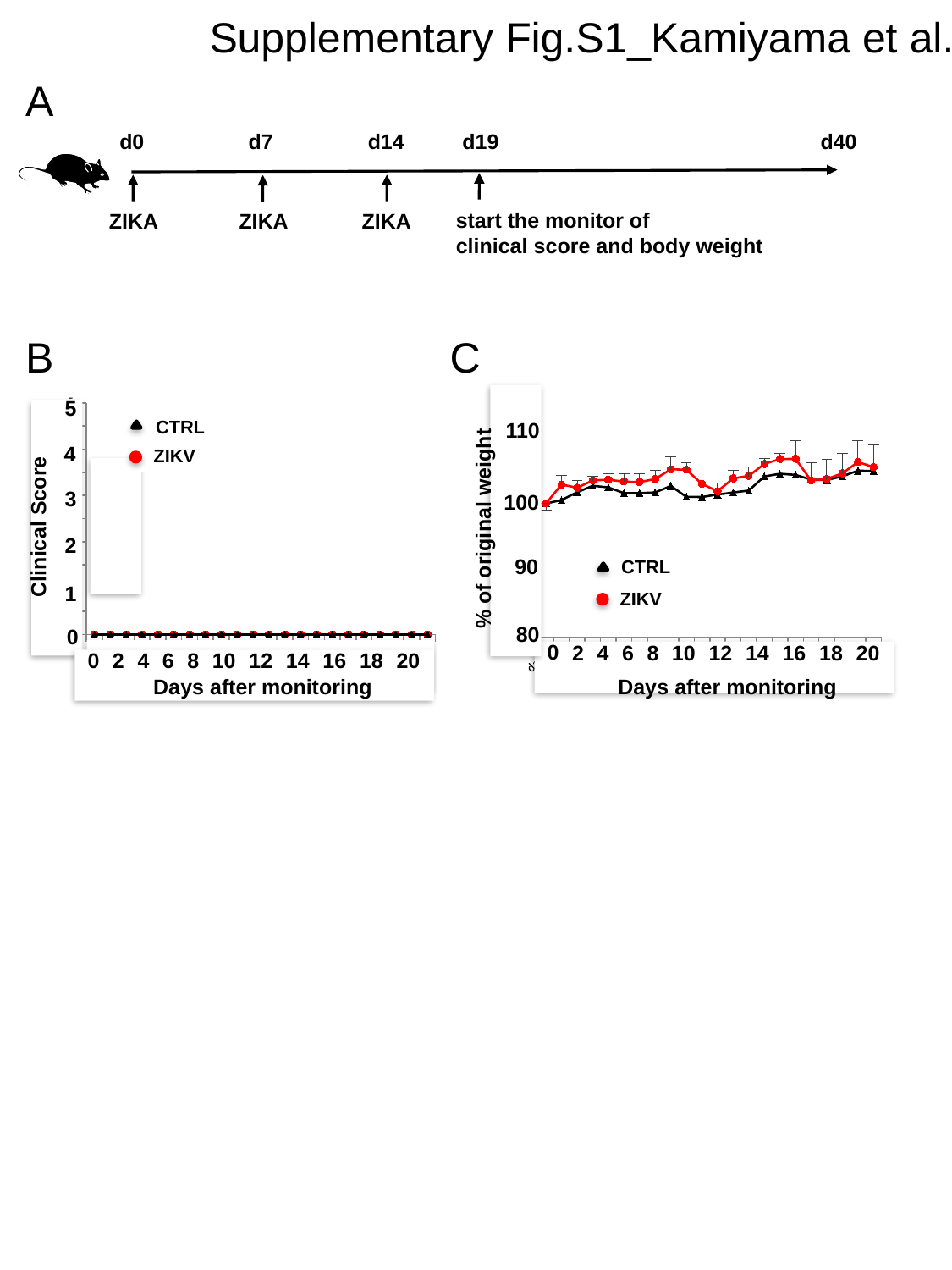

Supplementary Fig.S1_Kamiyama et al.
A
d0
d7
d14
d19
d40
start the monitor of
clinical score and body weight
ZIKA
ZIKA
ZIKA
B
C
### Chart
| Category | ZIKV | (-) |
|---|---|---|
| day0 | 0.0 | 0.0 |
| day1 | 0.0 | 0.0 |
| day2 | 0.0 | 0.0 |
| day3 | 0.0 | 0.0 |
| day4 | 0.0 | 0.0 |
| day5 | 0.0 | 0.0 |
| day6 | 0.0 | 0.0 |
| day7 | 0.0 | 0.0 |
| day8 | 0.0 | 0.0 |
| day9 | 0.0 | 0.0 |
| day10 | 0.0 | 0.0 |
| day11 | 0.0 | 0.0 |
| day12 | 0.0 | 0.0 |
| day13 | 0.0 | 0.0 |
| day14 | 0.0 | 0.0 |
| day15 | 0.0 | 0.0 |
| day16 | 0.0 | 0.0 |
| day17 | 0.0 | 0.0 |
| day18 | 0.0 | 0.0 |
| day19 | 0.0 | 0.0 |
| day20 | 0.0 | 0.0 |
| day21 | 0.0 | 0.0 |
### Chart
| Category | (-) | ZIKV |
|---|---|---|
| day0 | 100.0 | 100.0 |
| day1 | 100.5335082310189 | 102.8522575303087 |
| day2 | 101.6885727463503 | 102.3660472128484 |
| day3 | 102.6629783647449 | 103.4656878827647 |
| day4 | 102.4192548041197 | 103.5697428446444 |
| day5 | 101.5629561377901 | 103.2882577177853 |
| day6 | 101.5735033894777 | 103.2178704224472 |
| day7 | 101.6588670427967 | 103.6791924446944 |
| day8 | 102.6452562068638 | 105.1266005811773 |
| day9 | 101.0034872689533 | 105.0804079177603 |
| day10 | 100.982149824431 | 102.9450529621297 |
| day11 | 101.3431217345852 | 101.8487978065242 |
| day12 | 101.6549838063607 | 103.7709583177103 |
| day13 | 101.9452654924666 | 104.1521341082365 |
| day14 | 104.0659973887316 | 105.9334958130234 |
| day15 | 104.4655291437651 | 106.6718144606924 |
| day16 | 104.2988009774277 | 106.6928837020372 |
| day17 | 103.5897104387457 | 103.4371289526309 |
| day18 | 103.519138860374 | 103.6842269716285 |
| day19 | 104.0788506703581 | 104.5301102987127 |
| day20 | 104.9290871075952 | 106.2242329083865 |
| day21 | 104.8774282981305 | 105.4494805336833 |5
CTRL
110
4
ZIKV
3
100
Clinical Score
% of original weight
2
90
CTRL
1
ZIKV
80
0
0
2
4
6
8
10
12
14
16
18
20
0
2
4
6
8
10
12
14
16
18
20
Days after monitoring
Days after monitoring
