# Supplementary material for: Zika virus exacerbates encephalomyelitis by inducing the production of T cell-attracting chemokines in astrocytes
Source: Int Immunol. 2025 Dec 17;38(5):318–34. doi: 10.1093/intimm/dxaf075 (PMC13150445; doi:10.1093/intimm/dxaf075)
Supplement: dxaf075_Supplementary_Data [file dxaf075_supplementary_data.zip › Figure_International immunology FigureS10.pptx]

## Slide 1
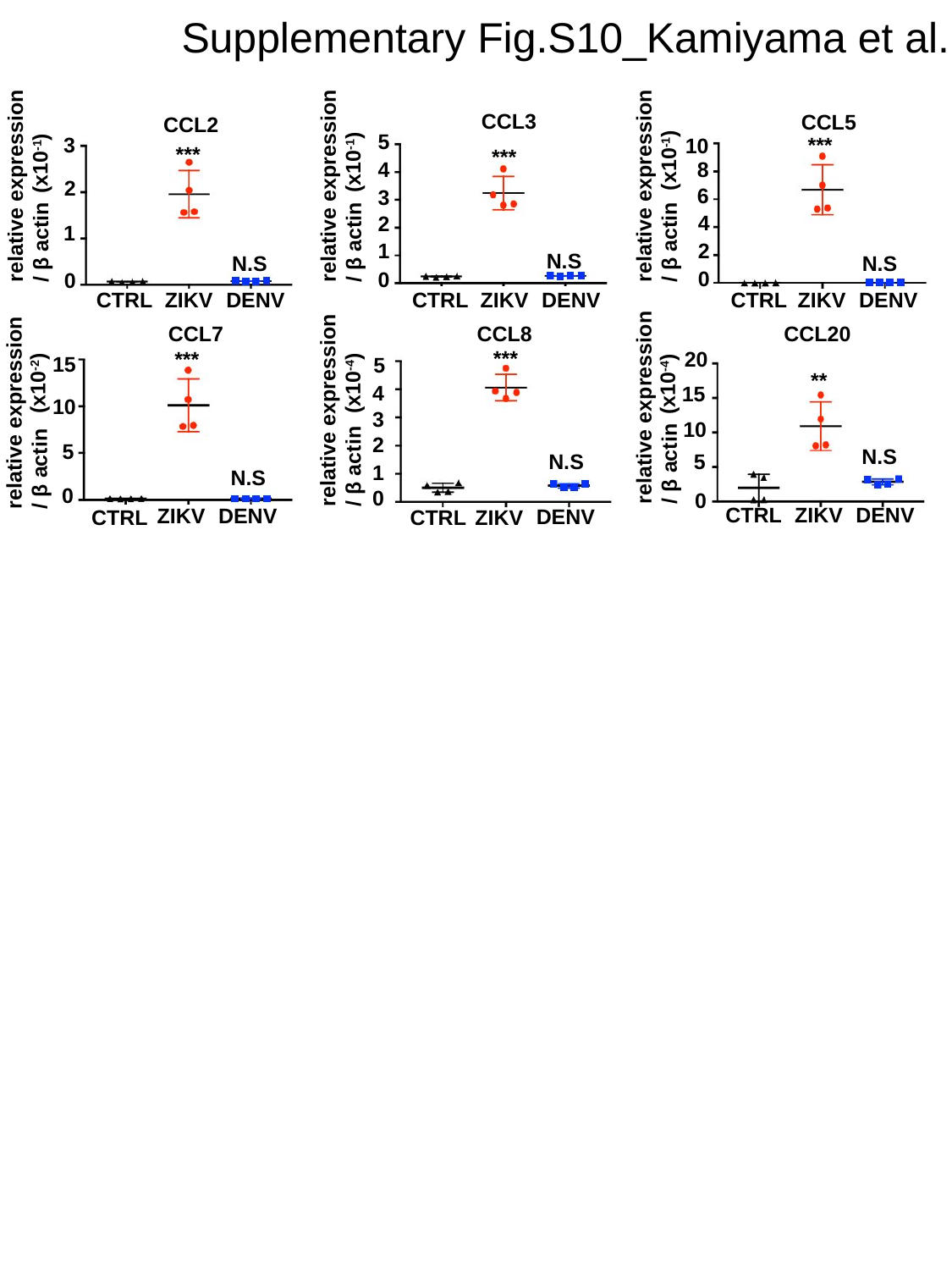

Supplementary Fig.S10_Kamiyama et al.
CCL3
CCL5
CCL2
5
3
***
10
***
***
(x10-1)
(x10-1)
(x10-1)
4
8
 relative expression
 / β actin
 relative expression
 / β actin
 relative expression
 / β actin
2
6
3
4
2
1
2
1
N.S
N.S
N.S
0
0
0
CTRL
ZIKV
DENV
CTRL
ZIKV
DENV
CTRL
ZIKV
DENV
CCL7
CCL8
CCL20
***
20
***
15
5
 relative expression
 / β actin
**
 relative expression
 / β actin
(x10-2)
(x10-4)
 relative expression
 / β actin
(x10-4)
4
15
10
3
10
2
5
N.S
N.S
5
1
N.S
0
0
0
DENV
ZIKV
CTRL
ZIKV
DENV
DENV
CTRL
ZIKV
CTRL
