# Supplementary material for: Zika virus exacerbates encephalomyelitis by inducing the production of T cell-attracting chemokines in astrocytes
Source: Int Immunol. 2025 Dec 17;38(5):318–34. doi: 10.1093/intimm/dxaf075 (PMC13150445; doi:10.1093/intimm/dxaf075)
Supplement: dxaf075_Supplementary_Data [file dxaf075_supplementary_data.zip › Figure_International immunology FigureS11.pptx]

## Slide 1
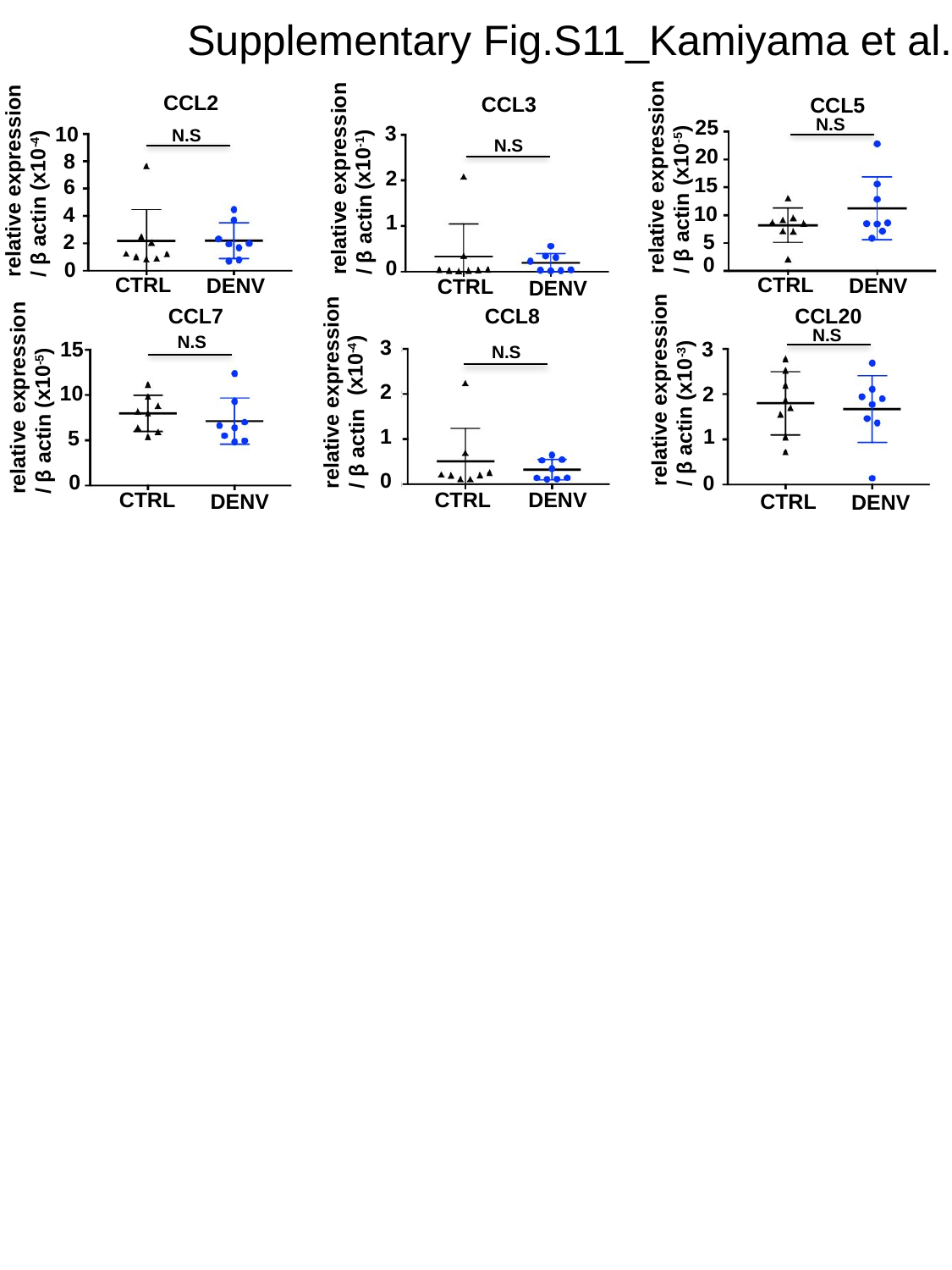

Supplementary Fig.S11_Kamiyama et al.
CCL2
CCL3
CCL5
N.S
25
3
10
N.S
N.S
 relative expression
 / β actin
 relative expression
 / β actin
 relative expression
 / β actin
(x10-5)
20
(x10-1)
(x10-4)
8
2
15
6
10
4
1
2
5
0
0
0
CTRL
CTRL
DENV
DENV
CTRL
DENV
CCL7
CCL8
CCL20
N.S
N.S
3
3
15
N.S
 relative expression
 / β actin
 relative expression
 / β actin
(x10-4)
 relative expression
 / β actin
(x10-3)
(x10-5)
2
10
2
1
1
5
0
0
0
CTRL
DENV
CTRL
DENV
CTRL
DENV
