# Supplementary material for: Zika virus exacerbates encephalomyelitis by inducing the production of T cell-attracting chemokines in astrocytes
Source: Int Immunol. 2025 Dec 17;38(5):318–34. doi: 10.1093/intimm/dxaf075 (PMC13150445; doi:10.1093/intimm/dxaf075)
Supplement: dxaf075_Supplementary_Data [file dxaf075_supplementary_data.zip › Figure_International immunology FigureS12.pptx]

## Slide 1
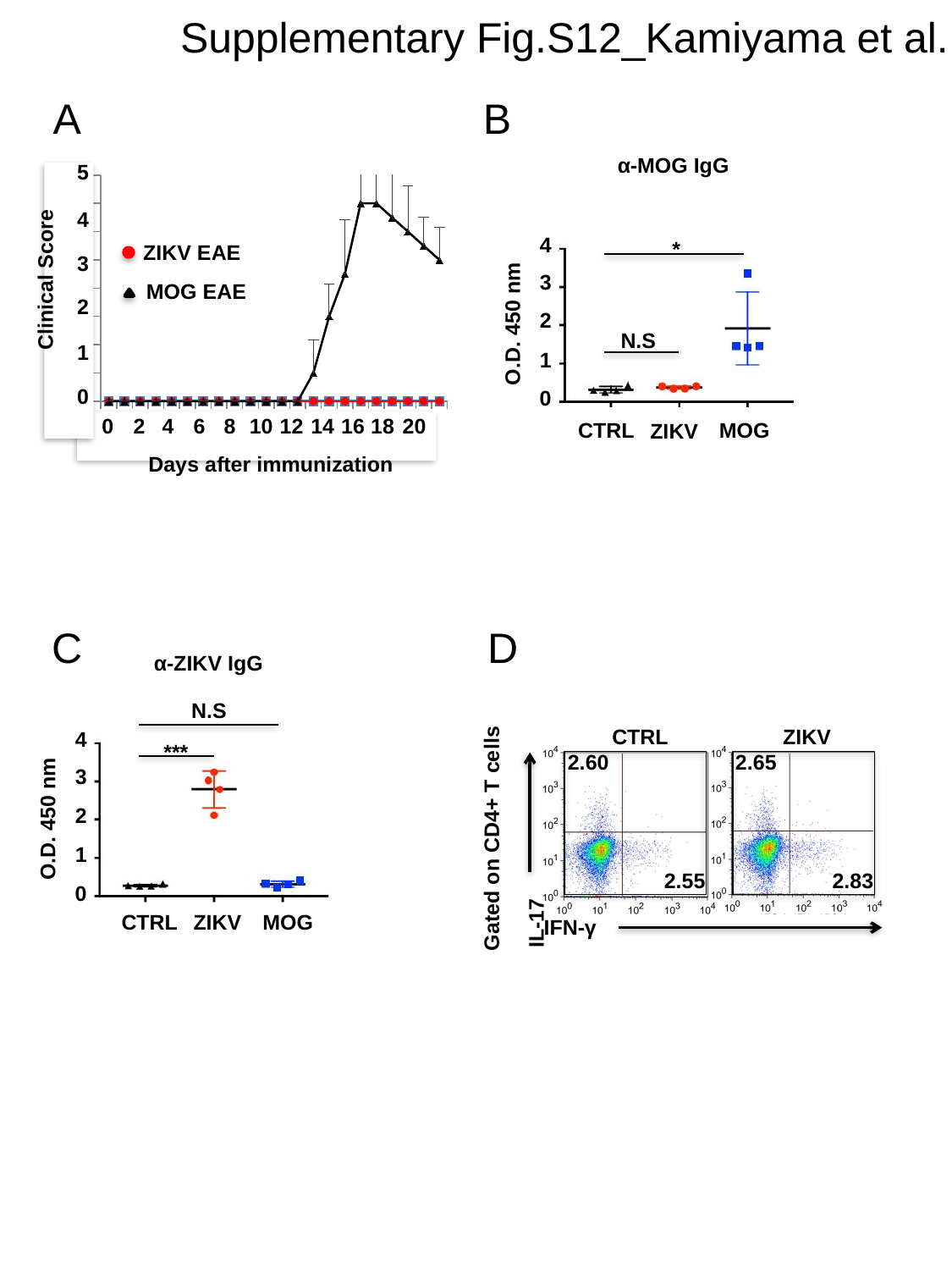

Supplementary Fig.S12_Kamiyama et al.
A
B
α-MOG IgG
5
### Chart
| Category | NC | ZIKV EAE | MOG EAE |
|---|---|---|---|
| day0 | 0.0 | 0.0 | 0.0 |
| day1 | 0.0 | 0.0 | 0.0 |
| day2 | 0.0 | 0.0 | 0.0 |
| day3 | 0.0 | 0.0 | 0.0 |
| day4 | 0.0 | 0.0 | 0.0 |
| day5 | 0.0 | 0.0 | 0.0 |
| day6 | 0.0 | 0.0 | 0.0 |
| day7 | 0.0 | 0.0 | 0.0 |
| day8 | 0.0 | 0.0 | 0.0 |
| day9 | 0.0 | 0.0 | 0.0 |
| day10 | 0.0 | 0.0 | 0.0 |
| day11 | 0.0 | 0.0 | 0.0 |
| day12 | 0.0 | 0.0 | 0.0 |
| day13 | 0.0 | 0.0 | 0.5 |
| day14 | 0.0 | 0.0 | 1.5 |
| day15 | 0.0 | 0.0 | 2.25 |
| day16 | 0.0 | 0.0 | 3.5 |
| day17 | 0.0 | 0.0 | 3.5 |
| day18 | 0.0 | 0.0 | 3.25 |
| day19 | 0.0 | 0.0 | 3.0 |
| day20 | 0.0 | 0.0 | 2.75 |
| day21 | 0.0 | 0.0 | 2.5 |4
4
*
ZIKV EAE
3
Clinical Score
3
MOG EAE
2
2
O.D. 450 nm
N.S
1
1
0
0
0
2
4
6
8
10
12
14
16
18
20
MOG
CTRL
ZIKV
Days after immunization
C
D
α-ZIKV IgG
N.S
 CTRL
ZIKV
4
***
2.60
2.65
3
2
O.D. 450 nm
Gated on CD4+ T cells
1
2.55
2.83
0
MOG
CTRL
ZIKV
IFN-γ
IL-17
