# Supplementary material for: Zika virus exacerbates encephalomyelitis by inducing the production of T cell-attracting chemokines in astrocytes
Source: Int Immunol. 2025 Dec 17;38(5):318–34. doi: 10.1093/intimm/dxaf075 (PMC13150445; doi:10.1093/intimm/dxaf075)
Supplement: dxaf075_Supplementary_Data [file dxaf075_supplementary_data.zip › Figure_International immunology FigureS13.pptx]

## Slide 1
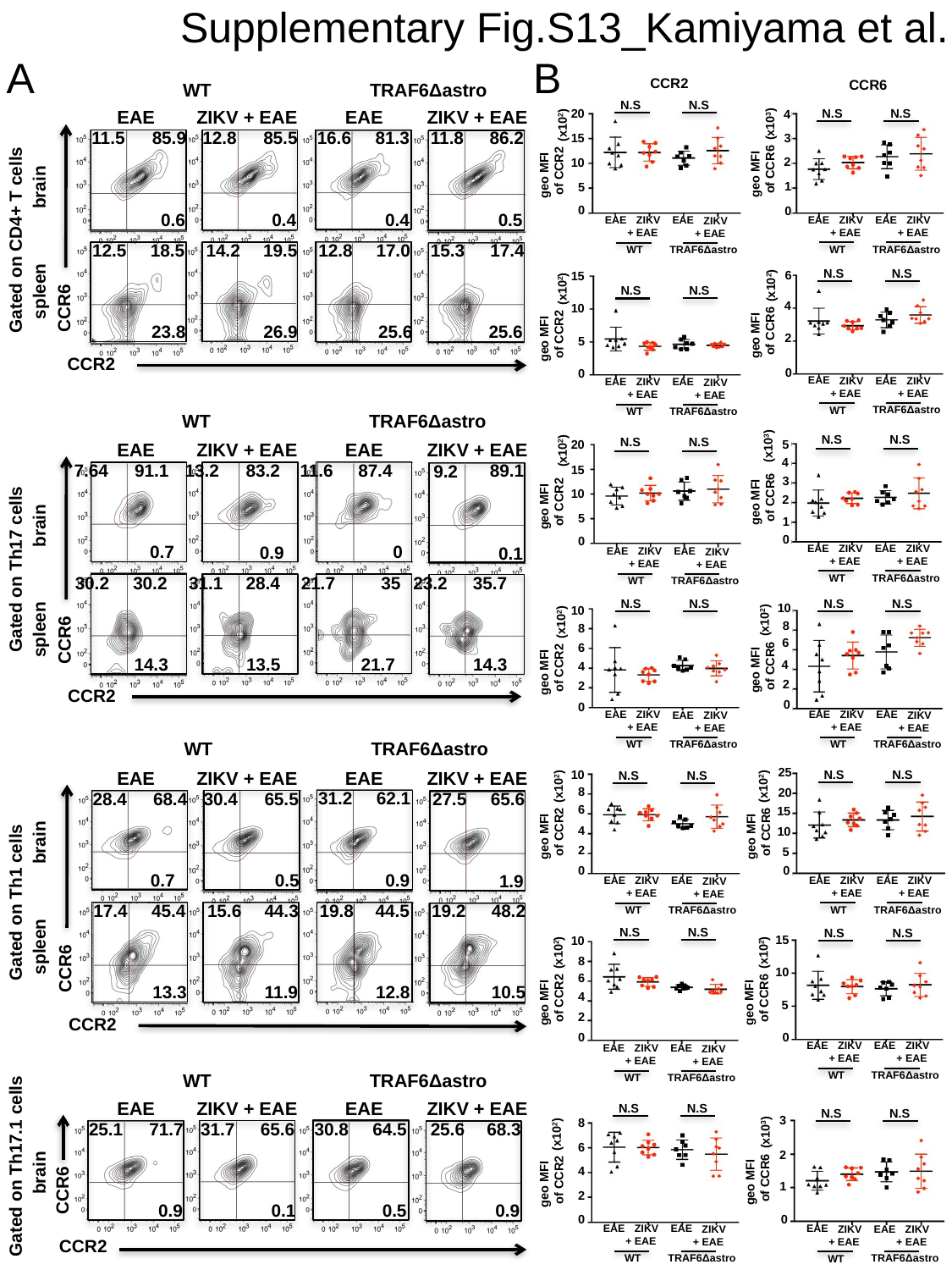

Supplementary Fig.S13_Kamiyama et al.
A
B
CCR2
CCR6
 WT
 TRAF6Δastro
N.S
N.S
20
 EAE
ZIKV + EAE
 EAE
ZIKV + EAE
N.S
N.S
4
(x103)
(x102)
11.5
85.9
12.8
85.5
16.6
81.3
11.8
86.2
3
15
geo MFI
 of CCR6
geo MFI
 of CCR2
2
10
brain
1
5
0
0
0.6
0.4
0.4
0.5
 EAE
 ZIKV
+ EAE
 EAE
 EAE
 ZIKV
+ EAE
 ZIKV
+ EAE
 EAE
 ZIKV
+ EAE
Gated on CD4+ T cells
12.5
18.5
14.2
19.5
12.8
17.0
15.3
17.4
 TRAF6Δastro
 WT
 TRAF6Δastro
 WT
N.S
N.S
6
15
spleen
(x102)
(x102)
N.S
N.S
CCR6
4
10
geo MFI
 of CCR6
geo MFI
 of CCR2
23.8
26.9
25.6
25.6
2
5
CCR2
0
0
 EAE
 ZIKV
+ EAE
 EAE
 ZIKV
+ EAE
 EAE
 ZIKV
+ EAE
 EAE
 ZIKV
+ EAE
 TRAF6Δastro
 WT
 TRAF6Δastro
 WT
 WT
 TRAF6Δastro
N.S
N.S
N.S
N.S
5
20
 EAE
ZIKV + EAE
 EAE
ZIKV + EAE
(x103)
(x102)
4
7.64
91.1
13.2
83.2
11.6
87.4
89.1
9.2
15
geo MFI
 of CCR6
3
geo MFI
 of CCR2
10
2
5
brain
1
0
0
0.7
0
0.9
 EAE
 ZIKV
+ EAE
 EAE
0.1
 ZIKV
+ EAE
 EAE
 ZIKV
+ EAE
 EAE
 ZIKV
+ EAE
Gated on Th17 cells
 TRAF6Δastro
 WT
30.2
30.2
31.1
28.4
21.7
35
23.2
35.7
 TRAF6Δastro
 WT
N.S
N.S
N.S
N.S
10
10
(x102)
spleen
(x102)
8
8
CCR6
6
geo MFI
 of CCR6
geo MFI
 of CCR2
6
14.3
13.5
21.7
14.3
4
4
2
2
CCR2
0
0
 EAE
 EAE
 ZIKV
+ EAE
 ZIKV
+ EAE
 EAE
 EAE
 ZIKV
+ EAE
 ZIKV
+ EAE
 WT
 TRAF6Δastro
 TRAF6Δastro
 TRAF6Δastro
 WT
 WT
25
N.S
10
N.S
 EAE
ZIKV + EAE
 EAE
ZIKV + EAE
N.S
N.S
(x102)
(x102)
8
20
31.2
62.1
27.5
30.4
65.5
65.6
28.4
68.4
6
15
geo MFI
 of CCR6
geo MFI
 of CCR2
4
10
brain
2
5
0
0
Gated on Th1 cells
0.7
0.9
0.5
1.9
 EAE
 ZIKV
+ EAE
 EAE
 EAE
 ZIKV
+ EAE
 ZIKV
+ EAE
 EAE
 ZIKV
+ EAE
17.4
45.4
15.6
44.3
19.8
44.5
19.2
48.2
 TRAF6Δastro
 WT
 TRAF6Δastro
 WT
N.S
N.S
N.S
N.S
spleen
15
10
(x102)
(x102)
CCR6
8
10
geo MFI
 of CCR6
geo MFI
 of CCR2
6
13.3
11.9
12.8
10.5
4
5
2
CCR2
0
0
 EAE
 ZIKV
+ EAE
 EAE
 ZIKV
+ EAE
 EAE
 ZIKV
+ EAE
 EAE
 ZIKV
+ EAE
 TRAF6Δastro
 WT
 WT
 TRAF6Δastro
 TRAF6Δastro
 WT
 EAE
ZIKV + EAE
 EAE
ZIKV + EAE
N.S
N.S
N.S
N.S
3
8
25.1
71.7
31.7
65.6
30.8
64.5
25.6
68.3
(x103)
(x102)
6
Gated on Th17.1 cells
2
geo MFI
 of CCR6
geo MFI
 of CCR2
brain
4
CCR6
1
2
0.9
0.1
0.5
0.9
0
0
 EAE
 ZIKV
+ EAE
 EAE
 EAE
 ZIKV
+ EAE
 ZIKV
+ EAE
 EAE
 ZIKV
+ EAE
CCR2
 TRAF6Δastro
 WT
 TRAF6Δastro
 WT
