# Supplementary material for: Zika virus exacerbates encephalomyelitis by inducing the production of T cell-attracting chemokines in astrocytes
Source: Int Immunol. 2025 Dec 17;38(5):318–34. doi: 10.1093/intimm/dxaf075 (PMC13150445; doi:10.1093/intimm/dxaf075)
Supplement: dxaf075_Supplementary_Data [file dxaf075_supplementary_data.zip › Figure_International immunology FigureS2.pptx]

## Slide 1
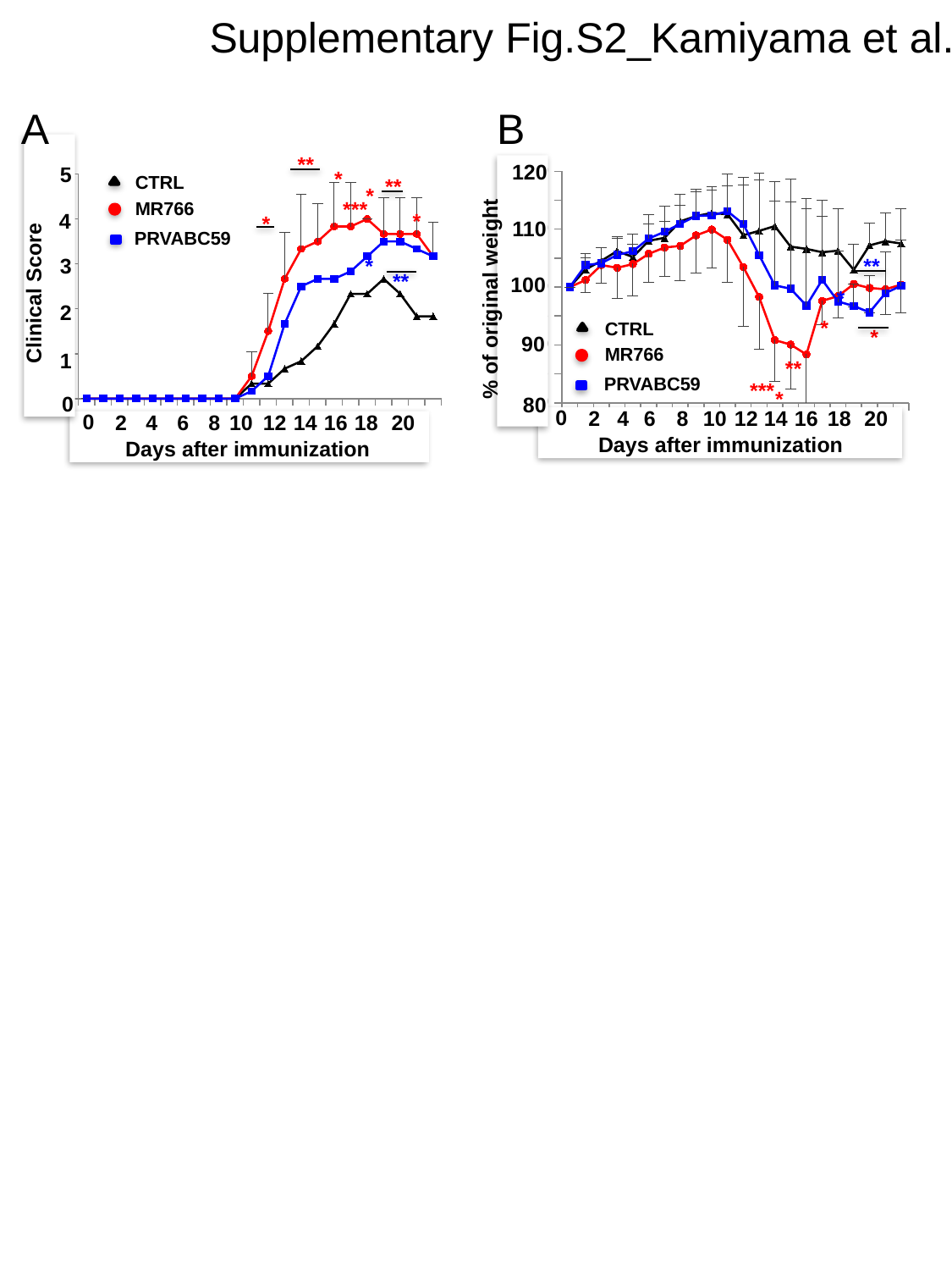

Supplementary Fig.S2_Kamiyama et al.
A
B
**
120
5
### Chart
| Category | (-) | MR | PRV |
|---|---|---|---|
| day0 | 100.0 | 100.0 | 100.0 |
| day1 | 103.0361267444674 | 101.2092829812854 | 103.7921094413312 |
| day2 | 104.540745467013 | 103.8115851364115 | 104.1264103295567 |
| day3 | 106.2144006038914 | 103.3347039714867 | 105.5546317692292 |
| day4 | 105.1952234220985 | 103.9747355044913 | 106.1929607293967 |
| day5 | 107.9905427694075 | 105.736828682694 | 108.3875201412251 |
| day6 | 108.5005180387102 | 106.8327661110033 | 109.5436217842564 |
| day7 | 111.3426480907641 | 107.1113958991937 | 110.9007887027783 |
| day8 | 112.2681794198265 | 108.929939278678 | 112.2860211748592 |
| day9 | 112.7928915409927 | 109.9367719640794 | 112.3886781796901 |
| day10 | 112.5776069997172 | 108.1674361777993 | 113.0910606752781 |
| day11 | 108.9532887401836 | 103.4765219680008 | 110.8946125308889 |
| day12 | 109.702431032216 | 98.28423404252764 | 105.5415965276239 |
| day13 | 110.5297704741028 | 90.84305419495621 | 100.2984822807784 |
| day14 | 106.9889338617551 | 90.0597910468275 | 99.71535433943795 |
| day15 | 106.5951161769492 | 88.36512183153248 | 96.80015929815174 |
| day16 | 106.0139063663066 | 97.62961236817138 | 101.2506443825287 |
| day17 | 106.2929419937002 | 98.4348686410481 | 97.51575350323971 |
| day18 | 102.9757006591415 | 100.5244403590698 | 96.73400002374306 |
| day19 | 107.2032989314718 | 99.85056280960808 | 95.63119335809013 |
| day20 | 107.9238083435155 | 99.62654283344453 | 98.95077243077904 |
| day21 | 107.5288003494015 | 100.4175410162467 | 100.237234702661 |*
CTRL
**
### Chart
| Category | (-) | MR | PRV |
|---|---|---|---|
| day0 | 0.0 | 0.0 | 0.0 |
| day1 | 0.0 | 0.0 | 0.0 |
| day2 | 0.0 | 0.0 | 0.0 |
| day3 | 0.0 | 0.0 | 0.0 |
| day4 | 0.0 | 0.0 | 0.0 |
| day5 | 0.0 | 0.0 | 0.0 |
| day6 | 0.0 | 0.0 | 0.0 |
| day7 | 0.0 | 0.0 | 0.0 |
| day8 | 0.0 | 0.0 | 0.0 |
| day9 | 0.0 | 0.0 | 0.0 |
| day10 | 0.333333333333333 | 0.5 | 0.166666666666667 |
| day11 | 0.333333333333333 | 1.5 | 0.5 |
| day12 | 0.666666666666667 | 2.666666666666666 | 1.666666666666667 |
| day13 | 0.833333333333333 | 3.333333333333333 | 2.5 |
| day14 | 1.166666666666667 | 3.5 | 2.666666666666666 |
| day15 | 1.666666666666667 | 3.833333333333333 | 2.666666666666666 |
| day16 | 2.333333333333333 | 3.833333333333333 | 2.833333333333333 |
| day17 | 2.333333333333333 | 4.0 | 3.166666666666666 |
| day18 | 2.666666666666666 | 3.666666666666666 | 3.5 |
| day19 | 2.333333333333333 | 3.666666666666666 | 3.5 |
| day20 | 1.833333333333333 | 3.666666666666666 | 3.333333333333333 |
| day21 | 1.833333333333333 | 3.166666666666666 | 3.166666666666666 |*
***
MR766
4
*
*
110
PRVABC59
3
*
**
**
100
Clinical Score
*
% of original weight
2
*
CTRL
*
90
MR766
1
**
PRVABC59
***
*
0
80
0
2
4
6
8
10
12
14
16
18
20
0
2
4
6
8
10
12
14
16
18
20
Days after immunization
Days after immunization
