# Supplementary material for: Zika virus exacerbates encephalomyelitis by inducing the production of T cell-attracting chemokines in astrocytes
Source: Int Immunol. 2025 Dec 17;38(5):318–34. doi: 10.1093/intimm/dxaf075 (PMC13150445; doi:10.1093/intimm/dxaf075)
Supplement: dxaf075_Supplementary_Data [file dxaf075_supplementary_data.zip › Figure_International immunology FigureS4.pptx]

## Slide 1
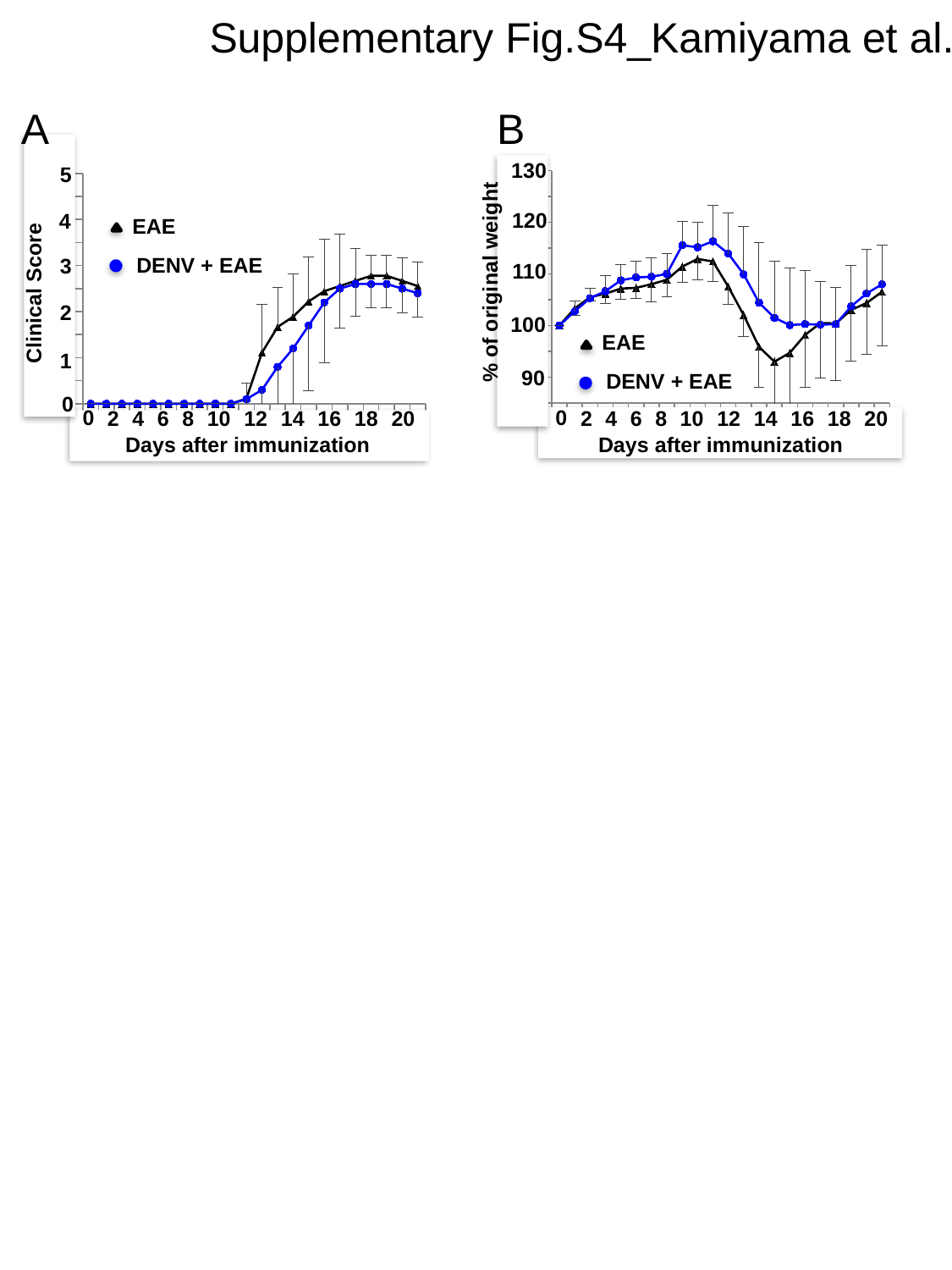

Supplementary Fig.S4_Kamiyama et al.
A
B
130
### Chart
| Category | (-) | DENV |
|---|---|---|
| day0 | 100.0 | 100.0 |
| day1 | 103.313319939572 | 102.7711871888328 |
| day2 | 105.5119605603704 | 105.324145521265 |
| day3 | 106.1892151131053 | 106.6768459410075 |
| day4 | 107.1837314283212 | 108.7710038173118 |
| day5 | 107.3187925525552 | 109.3363667492413 |
| day6 | 108.0340803636899 | 109.4749256378688 |
| day7 | 108.8870663900384 | 110.019162244581 |
| day8 | 111.4429401811146 | 115.5732309648655 |
| day9 | 112.9141312459578 | 115.1737454365683 |
| day10 | 112.4371350560041 | 116.3352103294861 |
| day11 | 107.5519113913431 | 113.9646737550595 |
| day12 | 102.0561196445454 | 109.9401919457516 |
| day13 | 95.8872290738676 | 104.4675815988562 |
| day14 | 93.02209105174597 | 101.5203348124311 |
| day15 | 94.71290050737873 | 100.1007270819769 |
| day16 | 98.2150454791052 | 100.290556233178 |
| day17 | 100.4840008301858 | 100.1791256176838 |
| day18 | 100.441964258375 | 100.286484924146 |
| day19 | 103.0388601143583 | 103.7297244982001 |
| day20 | 104.3732342283086 | 106.2277337487839 |
| day21 | 106.5499737617241 | 107.9983997661523 |
### Chart
| Category | (-) | DENV |
|---|---|---|
| day0 | 0.0 | 0.0 |
| day1 | 0.0 | 0.0 |
| day2 | 0.0 | 0.0 |
| day3 | 0.0 | 0.0 |
| day4 | 0.0 | 0.0 |
| day5 | 0.0 | 0.0 |
| day6 | 0.0 | 0.0 |
| day7 | 0.0 | 0.0 |
| day8 | 0.0 | 0.0 |
| day9 | 0.0 | 0.0 |
| day10 | 0.111111111111111 | 0.1 |
| day11 | 1.111111111111111 | 0.3 |
| day12 | 1.666666666666667 | 0.8 |
| day13 | 1.88888888888889 | 1.2 |
| day14 | 2.222222222222222 | 1.7 |
| day15 | 2.444444444444445 | 2.2 |
| day16 | 2.555555555555555 | 2.5 |
| day17 | 2.666666666666666 | 2.6 |
| day18 | 2.777777777777778 | 2.6 |
| day19 | 2.777777777777778 | 2.6 |
| day20 | 2.666666666666666 | 2.5 |
| day21 | 2.555555555555555 | 2.4 |5
120
4
EAE
DENV + EAE
3
110
% of original weight
Clinical Score
2
100
EAE
1
90
DENV + EAE
0
0
0
2
4
6
8
10
12
14
16
18
20
2
4
6
8
10
12
14
16
18
20
Days after immunization
Days after immunization
