# Supplementary material for: Zika virus exacerbates encephalomyelitis by inducing the production of T cell-attracting chemokines in astrocytes
Source: Int Immunol. 2025 Dec 17;38(5):318–34. doi: 10.1093/intimm/dxaf075 (PMC13150445; doi:10.1093/intimm/dxaf075)
Supplement: dxaf075_Supplementary_Data [file dxaf075_supplementary_data.zip › Figure_International immunology FigureS6.pptx]

## Slide 1
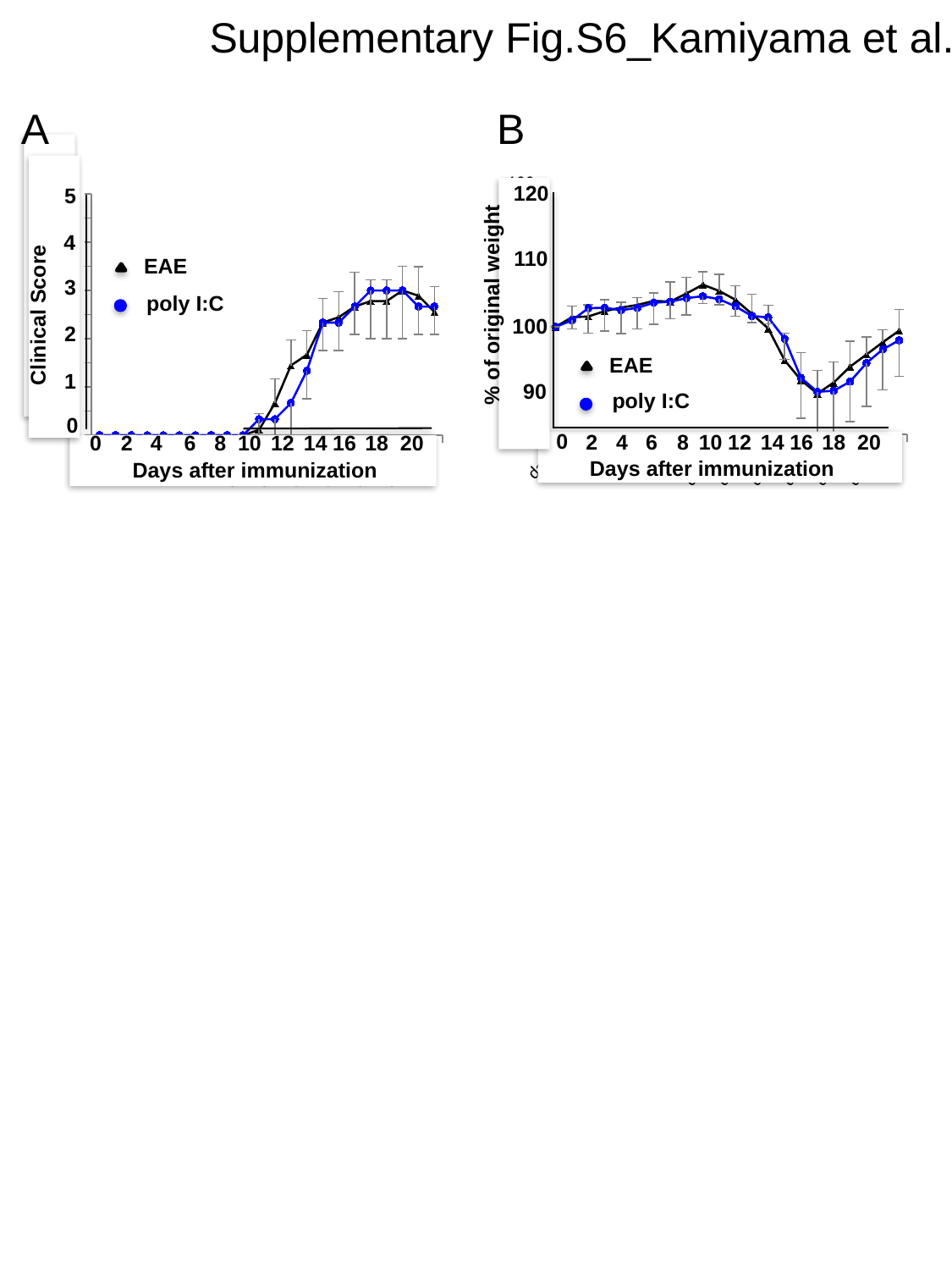

Supplementary Fig.S6_Kamiyama et al.
A
B
### Chart
| Category | (-) | polyIC |
|---|---|---|
| day0 | 100.0 | 100.0 |
| day1 | 101.3578587739629 | 100.911711961229 |
| day2 | 101.4536788080665 | 102.61408062315 |
| day3 | 102.1998482027412 | 102.6759425218855 |
| day4 | 102.6954626438715 | 102.3349278086375 |
| day5 | 103.0627523081718 | 102.6702384242418 |
| day6 | 103.6344524710002 | 103.3710797124153 |
| day7 | 103.5258579535339 | 103.5204162573111 |
| day8 | 104.6730665052748 | 104.0141384910697 |
| day9 | 105.8930689075337 | 104.2850825683219 |
| day10 | 105.017236028808 | 103.8648141856049 |
| day11 | 103.7826567913357 | 102.8639219008692 |
| day12 | 101.8226427035492 | 101.5327257237559 |
| day13 | 99.7764469485781 | 101.3233778895245 |
| day14 | 95.33028464706383 | 98.33750943281647 |
| day15 | 92.57794235518438 | 92.8810796051884 |
| day16 | 90.67889974736647 | 90.9386025028031 |
| day17 | 92.22348523746786 | 91.08300357349728 |
| day18 | 94.4399442803493 | 92.3545747606076 |
| day19 | 96.14202640866095 | 94.9490049722579 |
| day20 | 97.85451007678586 | 96.8591646983407 |
| day21 | 99.47478028673784 | 98.12896756883299 |120
### Chart
| Category | (-) | polyIC |
|---|---|---|
| day0 | 0.0 | 0.0 |
| day1 | 0.0 | 0.0 |
| day2 | 0.0 | 0.0 |
| day3 | 0.0 | 0.0 |
| day4 | 0.0 | 0.0 |
| day5 | 0.0 | 0.0 |
| day6 | 0.0 | 0.0 |
| day7 | 0.0 | 0.0 |
| day8 | 0.0 | 0.0 |
| day9 | 0.0 | 0.0 |
| day10 | 0.111111111111111 | 0.333333333333333 |
| day11 | 0.666666666666667 | 0.333333333333333 |
| day12 | 1.444444444444444 | 0.666666666666667 |
| day13 | 1.666666666666667 | 1.333333333333333 |
| day14 | 2.333333333333333 | 2.333333333333333 |
| day15 | 2.444444444444445 | 2.333333333333333 |
| day16 | 2.666666666666666 | 2.666666666666666 |
| day17 | 2.777777777777778 | 3.0 |
| day18 | 2.777777777777778 | 3.0 |
| day19 | 3.0 | 3.0 |
| day20 | 2.888888888888888 | 2.666666666666666 |
| day21 | 2.555555555555555 | 2.666666666666666 |5
4
110
EAE
3
poly I:C
% of original weight
Clinical Score
100
2
EAE
1
90
poly I:C
0
0
2
4
6
8
10
12
14
16
18
20
0
2
4
6
8
10
12
14
16
18
20
Days after immunization
Days after immunization
