# Supplementary figures and images for: Zika virus exacerbates encephalomyelitis by inducing the production of T cell-attracting chemokines in astrocytes
Source: Int Immunol. 2025 Dec 17;38(5):318–34. doi: 10.1093/intimm/dxaf075 (PMC13150445; doi:10.1093/intimm/dxaf075)

## Slide 1
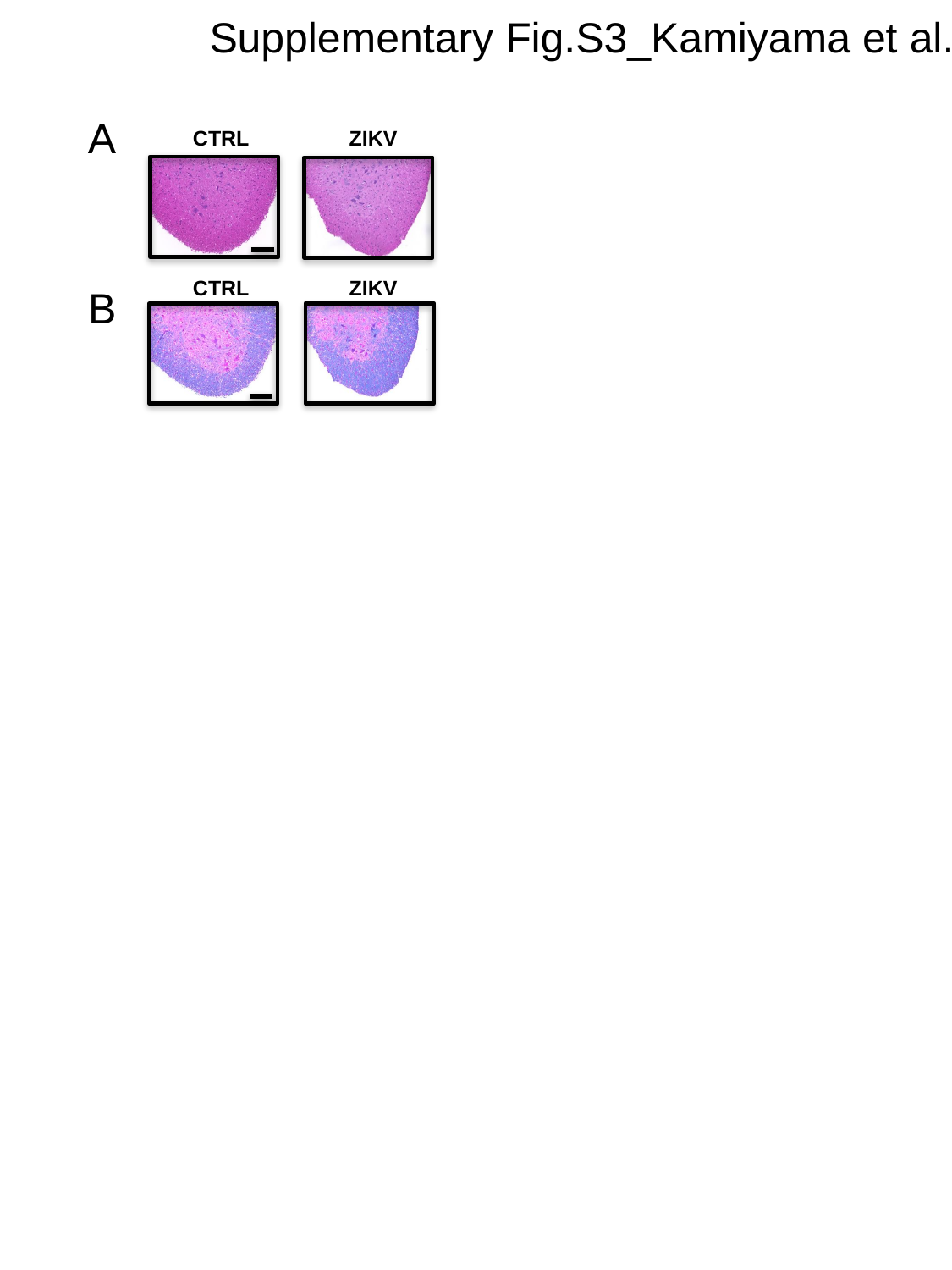

Supplementary Fig.S3_Kamiyama et al.
A
CTRL
ZIKV
CTRL
ZIKV
B

Supplement: dxaf075_Supplementary_Data [file dxaf075_supplementary_data.zip › Figure_International immunology FigureS3.pptx]

## Slide 1
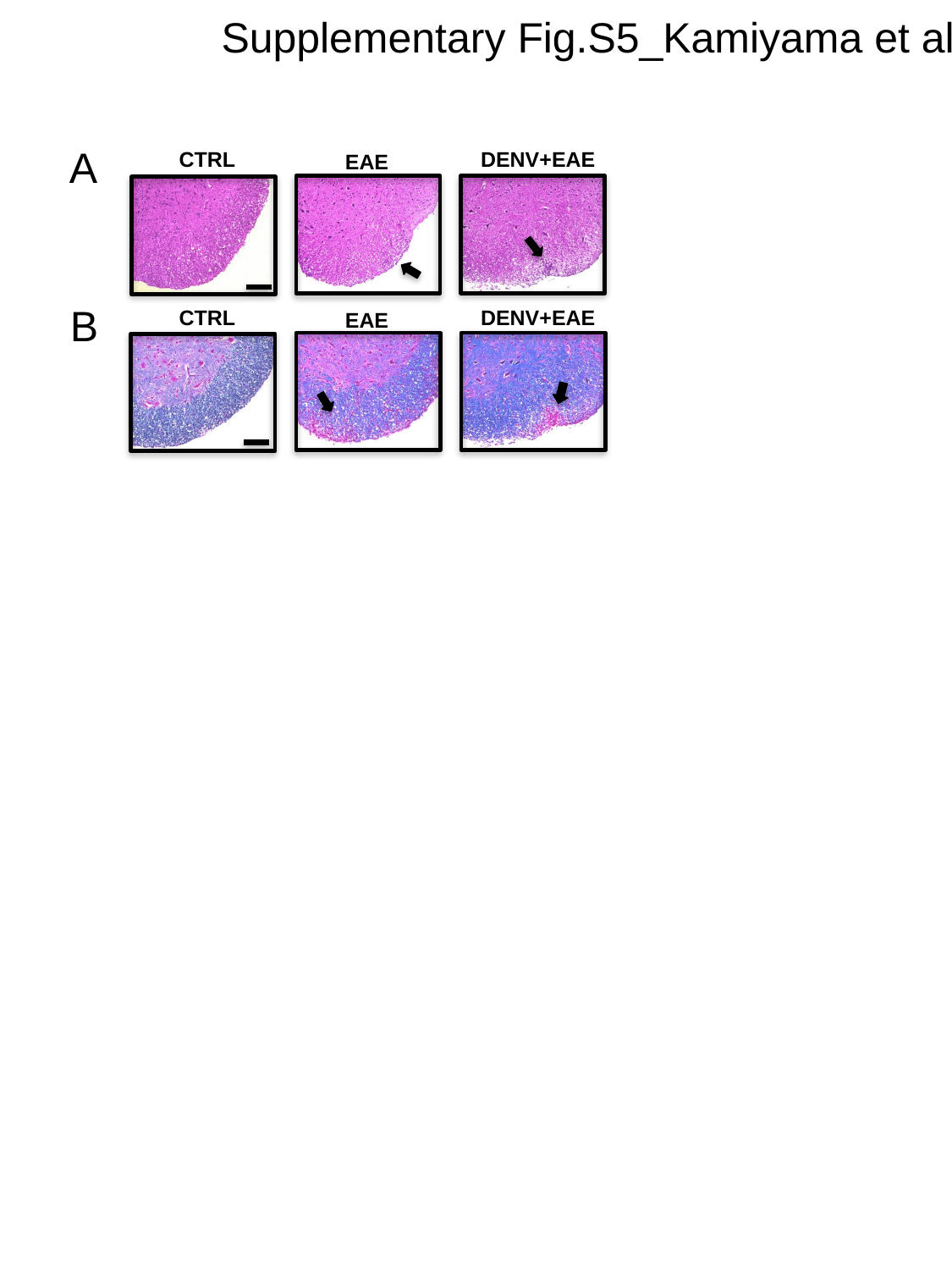

Supplementary Fig.S5_Kamiyama et al.
A
CTRL
DENV+EAE
EAE
B
CTRL
DENV+EAE
EAE

Supplement: dxaf075_Supplementary_Data [file dxaf075_supplementary_data.zip › Figure_International immunology FigureS5.pptx]

## Slide 1
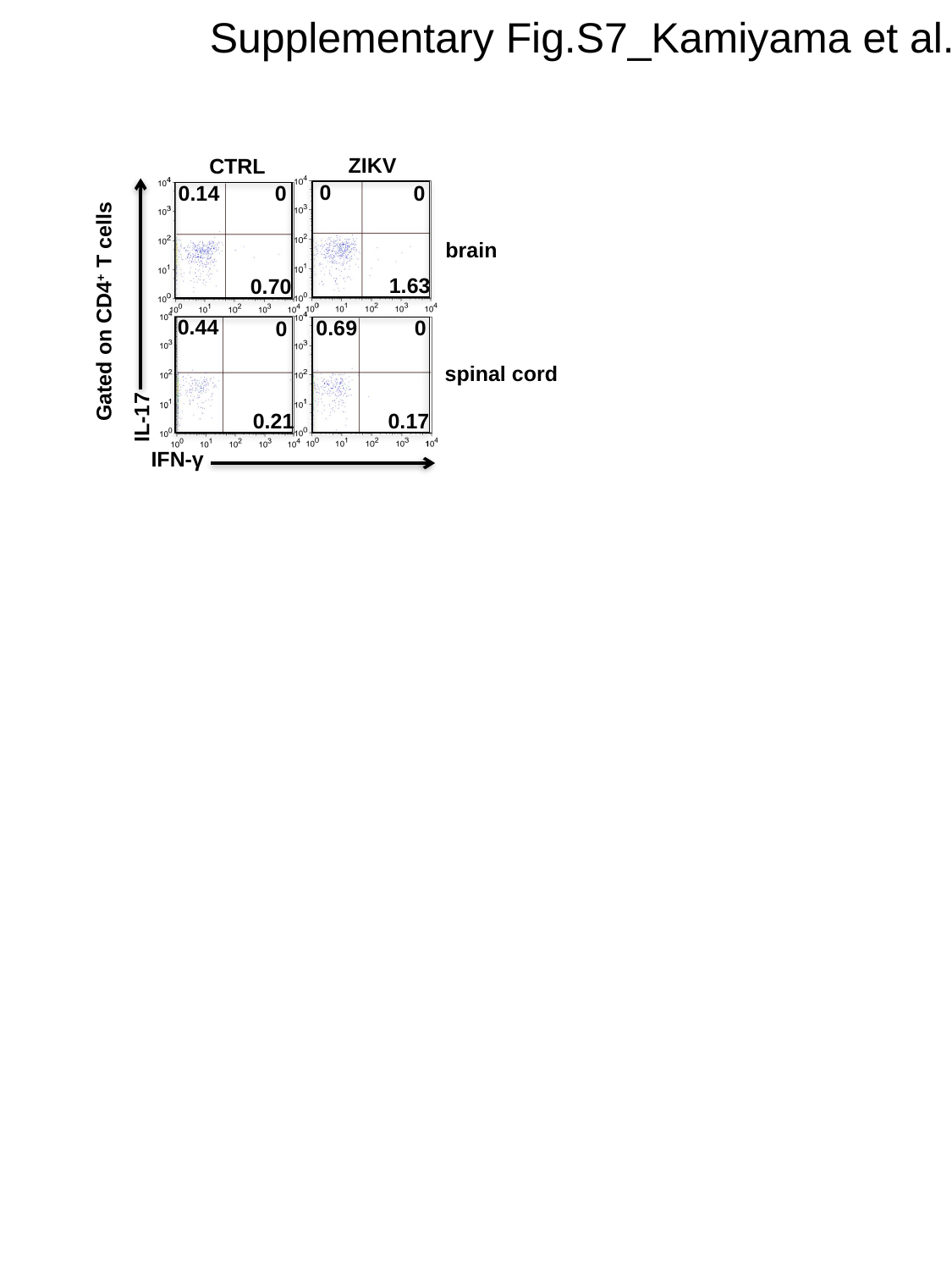

Supplementary Fig.S7_Kamiyama et al.
ZIKV
CTRL
0
0.14
0
0
brain
1.63
0.70
Gated on CD4+ T cells
0.44
0
0.69
0
spinal cord
IL-17
0.17
0.21
IFN-γ

Supplement: dxaf075_Supplementary_Data [file dxaf075_supplementary_data.zip › Figure_International immunology FigureS7.pptx]

## Slide 1
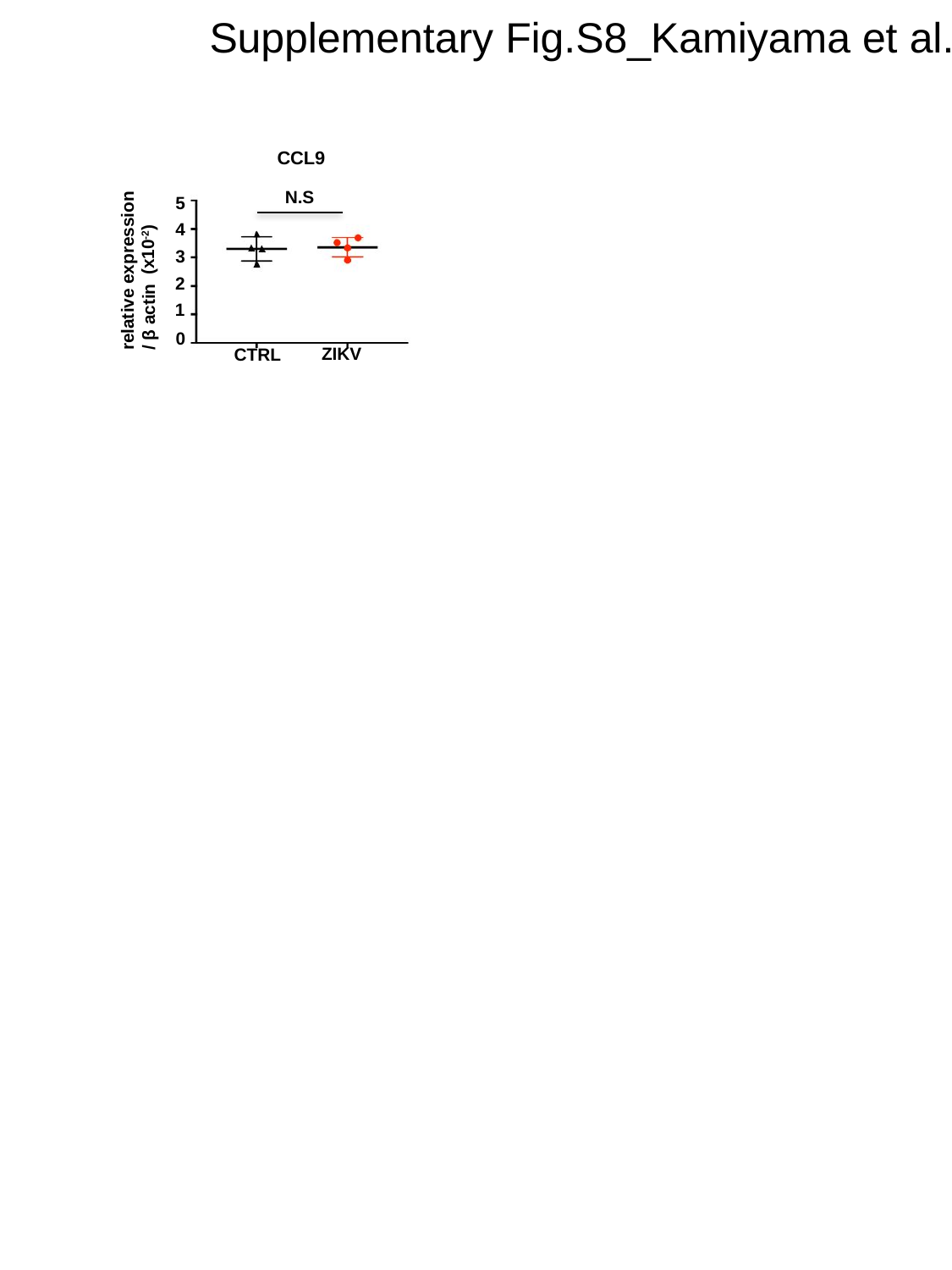

Supplementary Fig.S8_Kamiyama et al.
CCL9
N.S
5
4
(x10-2)
 relative expression
 / β actin
3
2
1
0
ZIKV
CTRL

Supplement: dxaf075_Supplementary_Data [file dxaf075_supplementary_data.zip › Figure_International immunology FigureS8.pptx]

## Slide 1
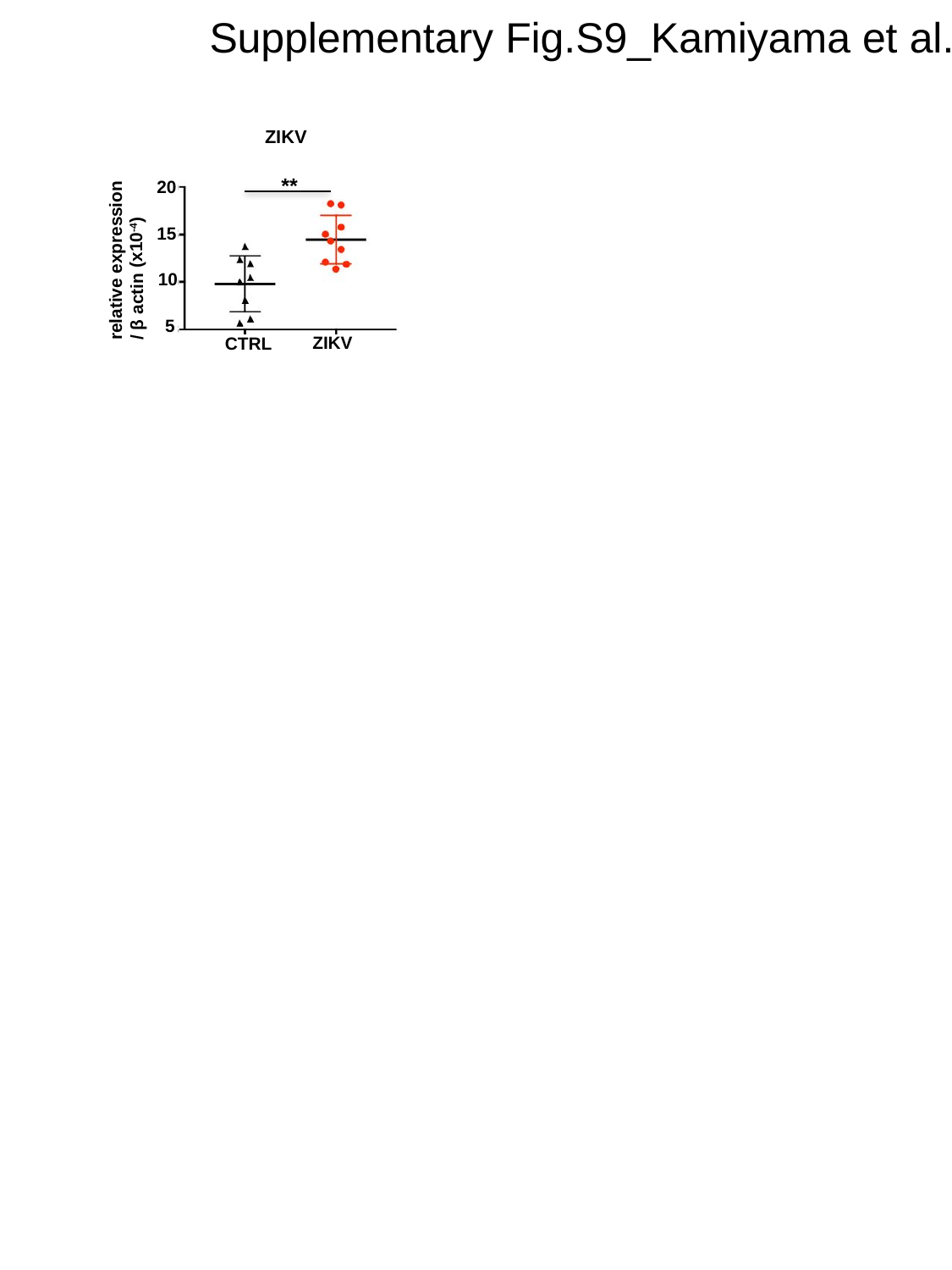

Supplementary Fig.S9_Kamiyama et al.
ZIKV
**
20
15
(x10-4)
 relative expression
 / β actin
10
5
ZIKV
CTRL

Supplement: dxaf075_Supplementary_Data [file dxaf075_supplementary_data.zip › Figure_International immunology FigureS9.pptx]
